# *Chlamydomonas* γ-tubulin mutations reveal a critical role for γ-TuRC in maintaining the stability of centriolar microtubules

**DOI:** 10.1242/bio.062609

**Published:** 2026-10-30

**Authors:** Yuki Nakazawa, Naoto Kubota, Mao Horii, Akira Noga, Yoshikazu Koike, Hiroko Kawai-Toyooka, Hideo Dohra, Katsushi Yamaguchi, Shuji Shigenobu, Ken-ichi Wakabayashi, Masafumi Hirono

**Affiliations:** ^1^ Department of Frontier Bioscience, Hosei University, Tokyo 184-8584, Japan; ^2^ Science and Technology Group, Okinawa Institute of Science and Technology Graduate University, Okinawa 904-2245, Japan; ^3^ Graduate School of Science and Engineering, Hosei University, Koganei, Tokyo 184-8584, Japan; ^4^ Department of Biological Science, Graduate School of Science, University of Tokyo, Tokyo 113-0033, Japan; ^5^ Research Institute of Green Science and Technology, Shizuoka University, Shizuoka 422-8529, Japan; ^6^ Shizuoka Instrumental Analysis Center, Shizuoka University, Shizuoka 422-8529, Japan; ^7^ Trans-Scale Biology Center, National Institute for Basic Biology, Aichi 444-8585, Japan; ^8^ Department of Industrial Life Sciences, Faculty of Life Sciences, Kyoto Sangyo University, Kyoto 603-8555, Japan

**Keywords:** Centriole, Triplet microtubule, γ-Tubulin, γ-TuRC, *Chlamydomonas*

## Abstract

The centriolar triplet microtubule consists of an A-tubule with 13 protofilaments, and B- and C-tubules, each with ten protofilaments. Although the formation of the triplets has been shown to require γ-tubulin, its specific role in the formation of each tubule remains unclear. We isolated two novel *Chlamydomonas reinhardtii* mutants, *bld13-1* and *bld13-2*, each expressing γ-tubulin with a single amino-acid substitution (T292I or E89D). Similar to known centriole-deficient mutants, both mutants exhibited defects in ciliary assembly, nuclear number, and the number and orientation of cytoplasmic microtubules. Genetic analyses of the mutants, along with the expression of the mutant γ-tubulins in the wild-type cells, suggested that both mutants exert dominant-negative effects over wild-type γ-tubulin. Interestingly, although the centrioles in these mutants retained the typical nine-triplet structure, their triplets frequently lacked several protofilaments in specific regions of the A- and C-tubules. The protofilament loss occurs more frequently in the proximal region of the centriole. These structural defects suggest a critical role for γ-tubulin in maintaining the stability of the A- and C-tubules of centriolar triplets.

## Teaser

**Summary:** Novel *Chlamydomonas* γ-tubulin mutations cause a partial loss of protofilaments in centriolar microtubules, indicating a critical role of γ-tubulin in structural stabilization of triplet microtubules.

## INTRODUCTION

Triplet microtubules (MTs) are a specialized type of MT found exclusively in centrioles, structures with ninefold symmetry that serve as the core of the centrosome and the basal bodies of cilia and flagella. Each triplet consists of an A-tubule, composed of 13 protofilaments arranged in a circle, and B- and C-tubules, each composed of ten protofilaments arranged in an arc. When centrioles function as basal bodies, the outer doublet MTs of the axoneme extend from the A- and B-tubules (Wang and Stearns, 2017; Osinka et al., 2019; LeGuennec et al., 2021). The unique structure of the centriolar triplet MTs is highly conserved across eukaryotic organisms; however, their assembly mechanism remains poorly understood (Azimzadeh and Marshall, 2010; Wang and Stearns, 2017; Guichard et al., 2023).

Similar to the assembly of cytoplasmic MTs, the triplet assembly likely involves the γ-tubulin ring complex (γ-TuRC) (Guichard et al., 2010; Schweizer and Lüders, 2021; Mukhopadhyay et al., 2026), which is a major nucleator of tubulin polymerization (Oakley et al., 2015; Wu and Akhmanova, 2017). It is composed of 14 γ-tubulin molecules arranged in an incomplete ring, along with GCP2∼6, MZT1, and other proteins. This structure suggests that it serves as a template for MT nucleation, positioning each γ-tubulin at the minus end of a protofilament during nucleation (Liu et al., 2021; Thawani and Petry, 2021; Zupa et al., 2021; Sulimenko et al., 2022). In animal cells, numerous γ-TuRCs within the pericentriolar materials (PCMs) of the centrosome nucleate cytoplasmic MTs, enabling the centrosome to function as the MT-organizing center (MTOC) (Oakley et al., 2015; Wu and Akhmanova, 2017).

In fact, the γ-tubulin or γ-TuRC has been reported to be essential for centriole formation in many organisms (Ruiz et al., 1999; Shang et al., 2002; Dammermann et al., 2004; Gogendeau et al., 2011; Bahtz et al., 2012; Cota et al., 2017). By analogy to its role in cytoplasmic MT assembly, the γ-TuRC is thought to function in the nucleation of centriolar triplet MTs as well. Supporting this hypothesis, cryo-electron microscopy of isolated human centrosomes revealed a conical structure, likely a γ-TuRC, capping the proximal end of the A-tubule in the immature centriole (Guichard et al., 2010). However, the γ-TuRC's nucleation activity for centriolar MTs has not been directly demonstrated, and its contribution to triplet MT assembly and/or maintenance, particularly of the B- and C-tubules, remains unclear.

Besides its prominent localization to the PCM, γ-TuRC is also found in various centriolar sites: in the proximal region of the triplet MT wall, on the outer and inner (luminal) surfaces of the MT wall, and on the distal appendages (Silflow et al., 1999; Schweizer and Lüders, 2021; Kalbfuss and Gönczy, 2023; Laporte et al., 2024). While the specific function of γ-TuRC at each site is unclear, a recent study in human cells showed that the depletion of proteins that localize γ-TuRCs to the inner surfaces of the centriole MTs destabilizes the centrioles and impairs ciliary assembly (Schweizer et al., 2021). These findings suggest that the γ-TuRCs within the centriole lumen play a crucial role in maintaining centriole structural integrity.

In this study, we isolated two novel *Chlamydomonas reinhardtii* strains carrying missense mutations in the single-copy γ-tubulin gene, which appear to exert dominant-negative effects. These are the first viable mutants of the γ-tubulin gene identified in *C. reinhardtii*. Similar to previously reported centriole-deficient mutants, these mutants exhibited defects in ciliary assembly. Intriguingly, their centrioles frequently lacked some of the protofilaments in the A- and C-tubules of triplet MTs. These phenotypes, along with the predicted positions of the mutations within the γ-tubulin molecule, support the hypothesis that γ-tubulin functions as a γ-TuRC component that stabilizes triplet MTs.

## RESULTS

### Novel γ-tubulin mutants of *Chlamydomonas*

We isolated two independent *Chlamydomonas* strains that showed defects in ciliary assembly and nuclear segregation, which are phenotypes characteristic of known centriole-deficient mutants (Fig. 1A,B; Table 1) (Ehler et al., 1995; Dutcher and Trabuco, 1998; Matsuura et al., 2004; Hiraki et al., 2007; Nakazawa et al., 2007). As genetic analyses mapped these mutations to the same locus on Linkage Group VI (Chromosome 6), we named the mutants *bld13-1* and *bld13-2* (Fig. 1C). Further linkage analyses showed that the causal mutations exhibited no recombination with a genetic marker designed within the γ-tubulin gene; among 188 progenies examined, no recombinants were detected. Whole-genome sequencing of *bld13-1* and PCR-based sequencing of the *bld13-2* γ-tubulin gene identified point mutations that cause single-amino-acid substitutions (*bld13-1*, T292I; *bld13-2*, E89D) in the γ-tubulin gene, a single-copy gene in *Chlamydomonas* (Fig. 1D). The original amino acids at these mutation sites are widely conserved among organisms including single-cell organisms and humans (Fig. 1D). To infer the possible effects of these mutations on the γ-tubulin structure, we compared AlphaFold2-predicted models of the *bld13-1* and *bld13-2* γ-tubulins with those of the wild type (WT) (Fig. S1A) (Jumper et al., 2021). The models suggest no predominant changes in the molecular conformation, except for a slight change in a small region on the molecular surface of *bld13-1* γ-tubulin.

**Fig. 1.**
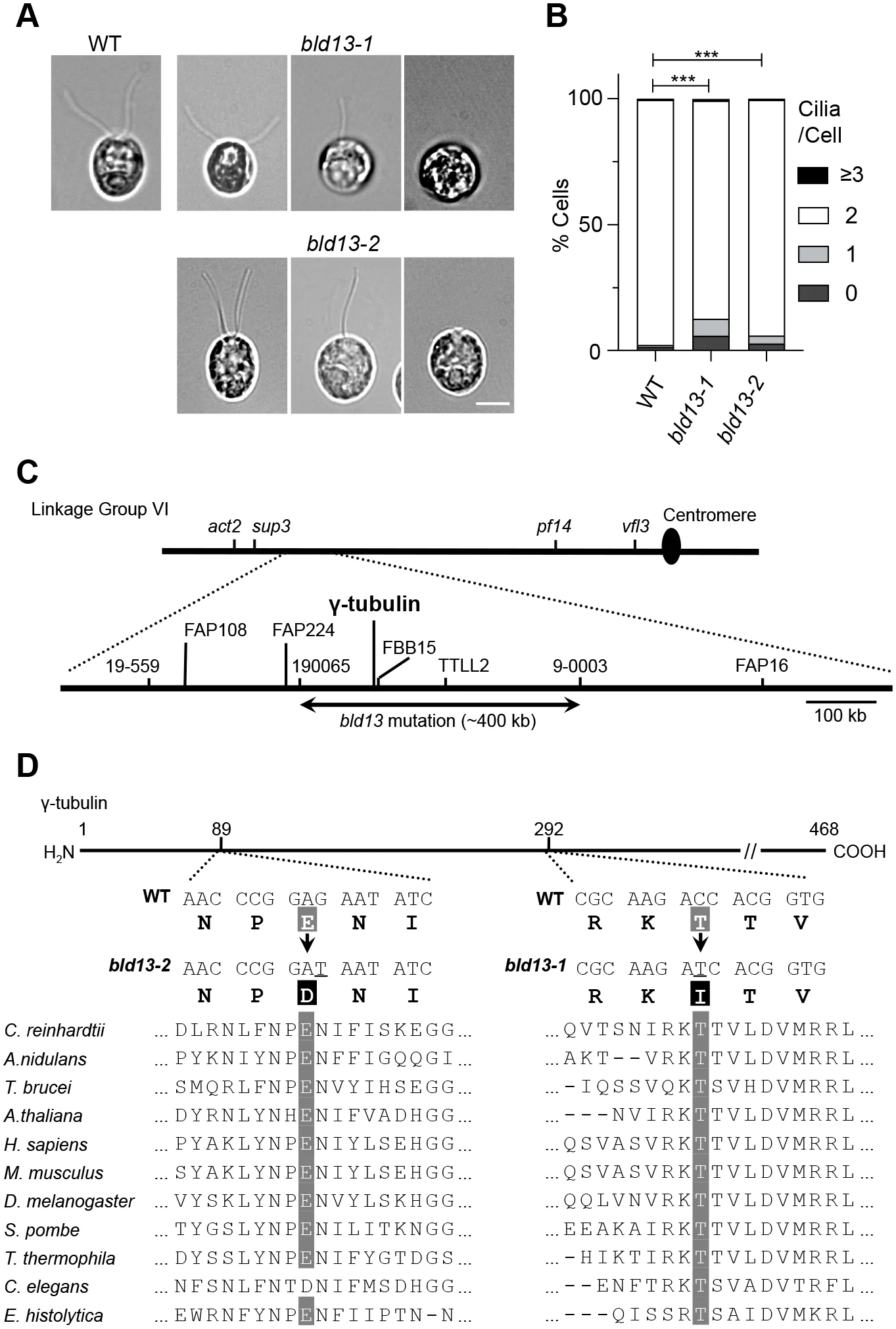
**Novel *Chlamydomonas* mutants *bld13-1* and *bld13-2*.** (A) Phase-contrast images of WT, *bld13-1* and *bld13-2* cells. Both *bld13* mutants contain cells with no or one cilium in addition to cells with two cilia. Scale bar: 5 μm. (B) Percentages of cells with zero, one, two, or three or more cilia in WT, *bld13-1*, and *bld13-2*. Total cell counts for each category are provided in Table S2, and variability between independent experiments is shown as dot plots in Fig. S2A. Differences in the distributions of cells across cilia-number categories between genotypes were assessed using Fisher's exact test on pooled cell counts (*n*>1500 cells per strain; ***P<0.001). (C) A genetic map showing a part of Linkage Group VI. The *bld13-1* and *bld13-2* mutations were mapped to an ∼400 kb region in this linkage group by AFLP-based analyses. The upper line shows the corresponding locus on the genetic map constructed by Kathir et al. (2003). The lower line shows positions of genes around this region (JGI Phytozome v 13, *Chlamydomonas* database v 5.6, https://phytozome-next.jgi.doe.gov/). Numbers 19-559, 190065, and 9-0003 represent primer sets used for PCR. FAP16, FAP108, and FAP224 are genes of flagellar-associated proteins (Pazour et al., 2005); FBB15 is a flagella/basal body protein gene (Li et al., 2004); and TTLL2 is a putative tubulin tyrosine ligase gene (Cre06.g300250, JGI Phytozome v 13, *Chlamydomonas* database v 5.6, https://phytozome-next.jgi.doe.gov/). (D) The positions of the *bld13-1* and *bld13-2* mutations in the γ-tubulin amino acid sequence of *Chlamydomonas* and corresponding sequences in other organisms. GenBank accession numbers are as follows: *C. reinhardtii*, AAA82610.1; *Aspergillus nidulans*, CAA33507.1; *Trypanosoma brucei*, XP_843687.1; *Arabidopsis thaliana*, NP_191724.1; *Homo sapiens*, NP_001061.2; *Mus musculus*, NP_598785.1; *Drosophila melanogaster*, NP_476804.1; *Schizosaccharomyces pombe*, NP_596147.1; *Tetrahymena thermophila*, AAB65830.1; *Caenorhabditis elegans*, NP_499131.1; *Entamoeba histolytica*, AAC08441.1.

**Table 1.** Abnormal nuclear segregation in *bld13* mutants

	Number of nuclei/cell			
Strain	0	1	2 or 3	Total
WT	14 (0.6)	2281 (99.3)	2 (0.1)	2297
bld12-1***	95 (3.7)	2318 (90.2)	157 (6.1)	2570
bld13-1***	28 (1.2)	2146 (95.3)	78 (3.5)	2252
bld13-2***	21 (0.6)	3247 (98.4)	32 (1.0)	3300

Values indicate the number of cells in each category (% in parentheses). Data present the total number of cells pooled from five independent experiments. Individual data counts for each experiment are provided in Table S1. *P*-values were calculated using Fisher's exact test for the overall distribution of nuclear numbers (***P<0.001 versus WT).

To computationally assess their functional impact, AlphaMissense was applied to human γ-tubulin (UniProt ID P23258) (Cheng et al., 2023). The equivalent E89D and T288I (corresponding to *Chlamydomonas* E89D and T292I, respectively) substitutions were both classified as ‘likely pathogenic’ (scores: 0.9508 and 0.9956, respectively).

### The *bld13-1* and *bld13-2* mutations are consistent with a dominant-negative mechanism

The defective phenotypes of the *bld13* mutants were not rescued by expression of a hemagglutinin (HA)-tagged γ-tubulin gene [γ-tub(WT)-HA]. In addition, expression of HA-tagged γ-tubulin carrying the *bld13-1* or *bld13-2* mutations in WT cells [WT::γ-tub(T292I)-HA and WT::γ-tub(E89D)-HA] significantly changed the proportions of cells with zero, one, two, or three or more cilia compared with WT::γ-tub(WT)-HA, indicating defects in ciliary assembly similar to those observed in the mutants (Fig. 2A,B). Together, these results suggest that the mutant γ-tubulins may interfere with WT γ-tubulin function, consistent with a dominant-negative effect. To test this possibility under equal gene dosage of WT and mutant alleles, we produced diploid strains by crossing the WT with *bld13-1* or *bld13-2* (WT/*bld13-1* and WT/*bld13-2*) and the *bld13-1*/*bld13-2* diploid. In these diploid strains carrying *bld13* mutations, the proportions of cells in each category were significantly shifted compared with those in the WT diploid (WT/WT), indicating defects in ciliary assembly similar to those observed in haploid mutants (Fig. 2C). These results support the interpretation that the *bld13-1* and *bld13-2* mutations interfere with WT γ-tubulin function in a dominant-negative manner.

**Fig. 2.**
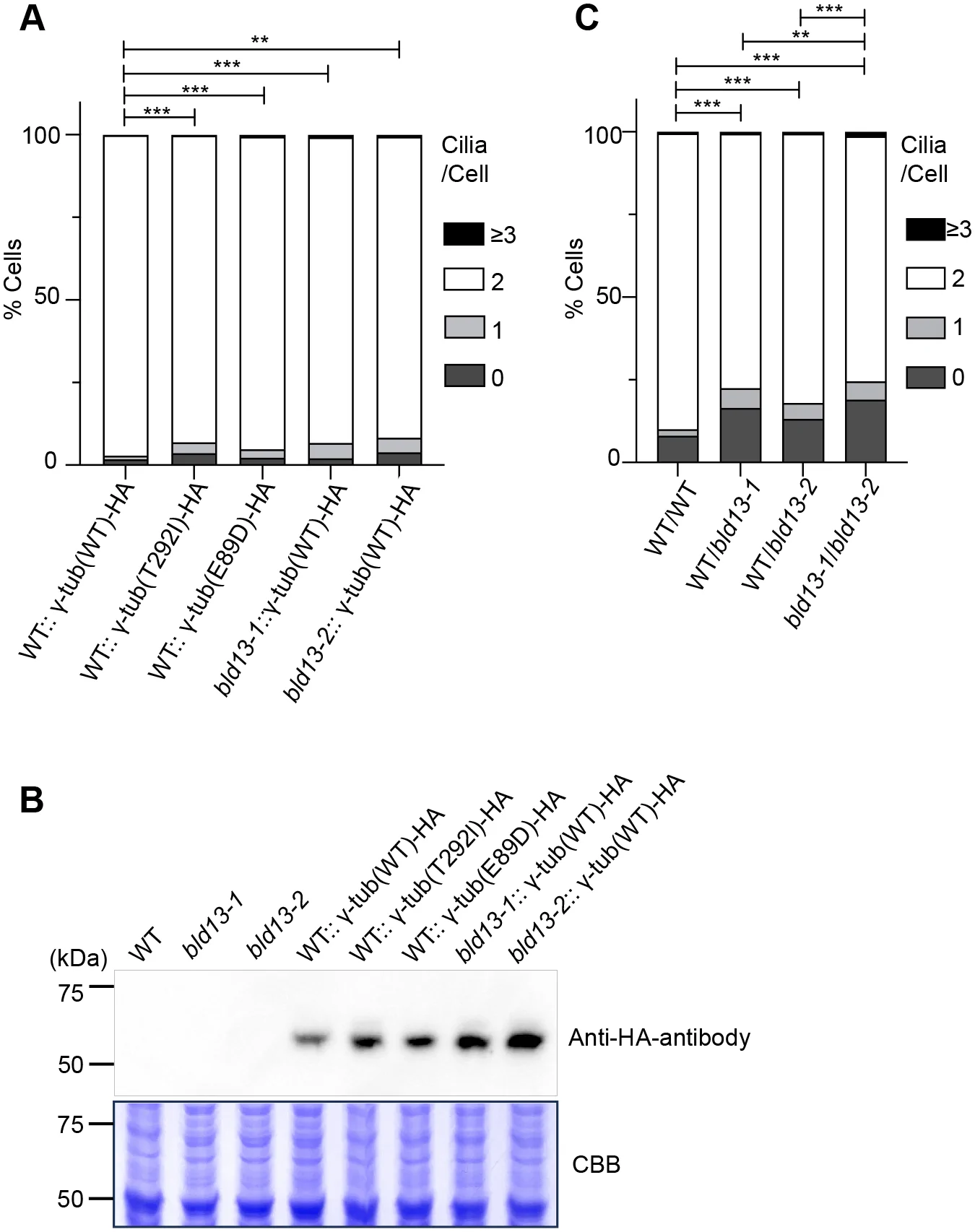
**Evidence supporting dominant-negative effects of the *bld13* mutations on ciliary assembly.** (A) Percentages of cells with zero, one, two, or three or more cilia in strains ectopically expressing HA-tagged γ-tubulin from genomic fragments under the control of the endogenous γ-tubulin promoter. These strains include a WT strain expressing an HA-tagged γ-tubulin [WT::γ-tub(WT)-HA], a WT strain expressing the *bld13-1* γ-tubulin [WT::γ-tub(T292I)-HA], a WT strain expressing the *bld13-2* γ-tubulin [WT::γ-tub(E89D)-HA], a *bld13-1* strain expressing the HA-tagged γ-tubulin [*bld13-1*::γ-tub(WT)-HA], and a *bld13-2* strain expressing the HA-tagged γ-tubulin [*bld13-2*::γ-tub(WT)-HA]. Total cell counts for each category are provided in Table S3, and variability between independent experiments is shown as dot plots in Fig. S2B. Differences in the distribution of cells across cilia-number categories between genotypes were assessed using Fisher's exact test on pooled cell counts (*n*>1500 cells per strain; **P<0.01, ***P<0.001). (B) Western blot analyses of whole-cell lysates (10 μg per lane) from the WT, *bld13-1*, *bld13-2*, WT::γ-tub(WT)-HA, WT::γ-tub(T292I)-HA, WT::γ-tub(E89D)-HA, *bld13-1*::γ-tub(WT)-HA, and *bld13-2*::γ-tub(WT)-HA cells. The upper panel shows immunoblotting with an anti-HA antibody detecting γ-tubulin(WT)-HA (55 kDa; arrowhead), and the lower panel shows Coomassie Brilliant Blue staining as a loading control. (C) Percentages of cells with zero, one, two, or three or more cilia in diploid strains: WT/WT, heterozygous *bld13* mutants (WT/*bld13-1* and WT/*bld13-2*), and the compound heterozygote *bld13-1*/*bld13-2*. Total cell counts for each category are provided in Table S4, and variability between independent experiments is shown as dot plots in Fig. S2C. Differences in the distribution of cells across cilia-number categories between genotypes were assessed as in A using Fisher's exact test on pooled cell counts (*n*>1500 cells per strain; **P<0.01, ***P<0.001).

### γ-Tubulin localizes to centrioles and their surrounding regions independently of *bld13* mutations

In the interphase *Chlamydomonas* cell, two mature centrioles (basal bodies) and two immature centrioles (probasal bodies) are present at the base of the two cilia, forming an organizing center for four stable rootlet MT bundles and non-rootlet cytoplasmic MTs (Fig. 3A; Holmes and Dutcher, 1989). SAS-6, a positional marker for centrioles, localizes to the proximal ends of both mature and immature centrioles, resulting in the observation of four discrete fluorescent spots.

**Fig. 3.**
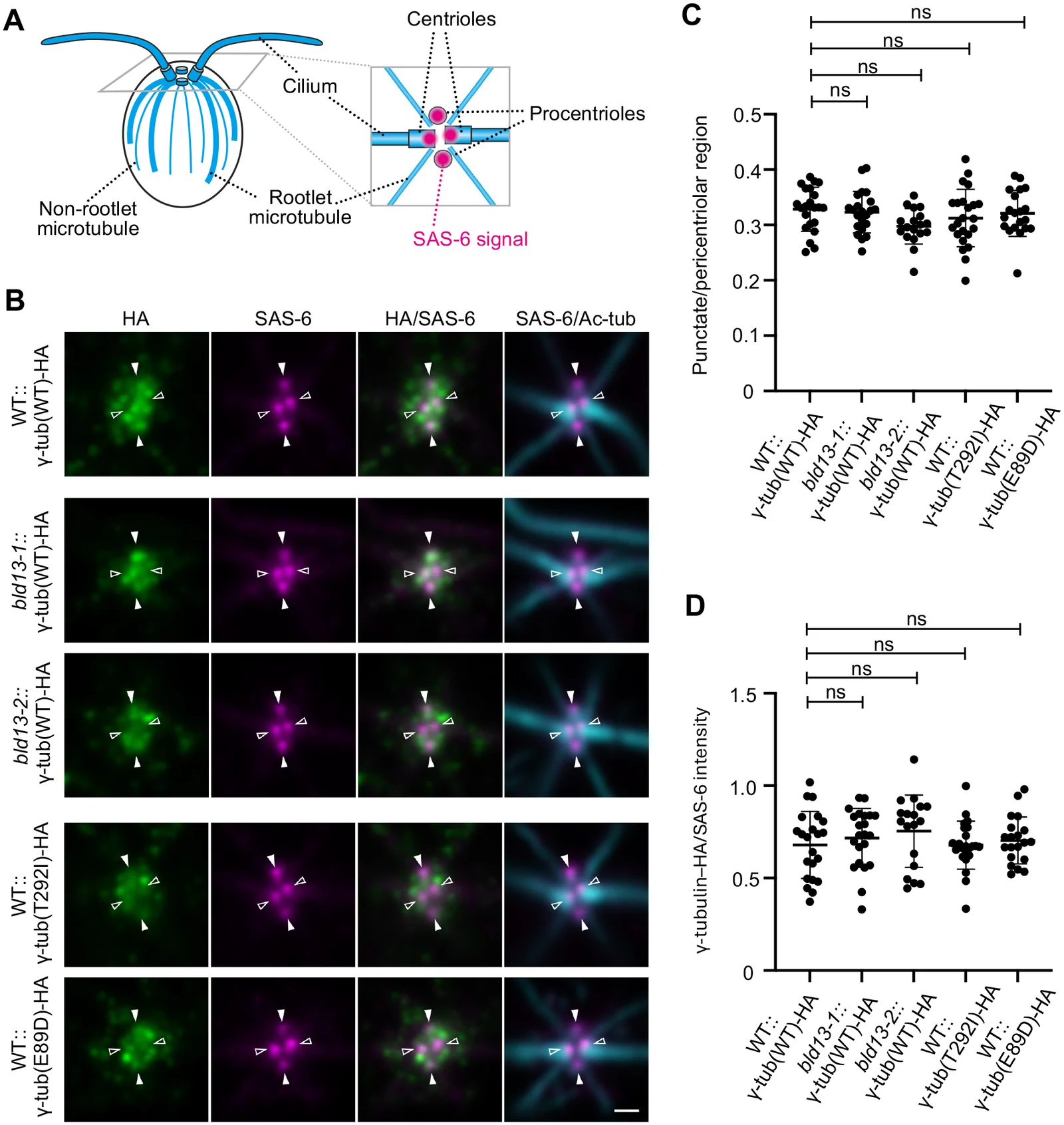
**Localization of γ-tubulin in the presence or absence of *bld13* mutations.** (A) Schematic diagram showing MT organization and centriole positioning in the *Chlamydomonas* cell. (B) Immunofluorescence images of WT::γ-tub(WT)-HA, *bld13-1*::γ-tub(WT)-HA, *bld13-2*::γ-tub(WT)-HA, WT::γ-tub(T292I)-HA, and WT::γ-tub(E89D)-HA cells stained with anti-HA (HA, green), anti-SAS-6 (SAS-6, magenta), and anti-acetylated tubulin (Ac-tub, cyan) antibodies. Merged images show HA and SAS-6 signals (HA/SAS-6) or SAS-6 and acetylated tubulin signals (SAS-6/Ac-tub). Scale bar: 500 nm. Filled arrowheads indicate procentrioles, and open arrowheads indicate mature centrioles. Representative images are shown; similar localization patterns were consistently observed. (C) Quantification of the fraction of punctate γ-tubulin-HA intensity within the total pericentriolar signal. Data are shown as individual values with mean±s.d. Differences between WT::γ-tub(WT)-HA and the other strains were assessed using one-way ANOVA followed by Dunnett's multiple-comparison test. *n*=22, 22, 18, 23, 20 cells for WT::γ-tub(WT)-HA, *bld13-1*::γ-tub(WT)-HA, *bld13-2*::γ-tub(WT)-HA, WT::γ-tub(T292I)-HA, and WT::γ-tub(E89D)-HA, respectively; ns, not significant (Dunnett-adjusted *P*≥0.05). Mean±s.d. values were 0.33±0.04, 0.32±0.04, 0.30±0.03, 0.31±0.05, and 0.32±0.04, respectively. (D) Quantification of γ-tubulin-HA at SAS-6-positive mature centrioles, expressed as the γ-tubulin-HA/SAS-6 fluorescence intensity ratio (see Materials and Methods). Data are shown as individual values with mean±s.d. Statistical analysis was performed as in C. *n*=22, 22, 18, 23, 20 cells, respectively; ns, not significant (Dunnett-adjusted *P*≥0.05). Mean±s.d. values were 0.68±0.18, 0.72±0.16, 0.75±0.20, 0.68±0.13, and 0.70±0.13, respectively. Data for C and D are from a single experiment, and the individual cells do not represent independent biological replicates.

In WT and *bld13* cells expressing HA-tagged WT γ-tubulin [Fig. 3B; WT::γ-tub(WT)-HA, *bld13-1*::γ-tub(WT)-HA, and *bld13-2*::γ-tub(WT)-HA], HA signals were detected in regions adjacent to the four centrioles, which are marked by SAS-6 fluorescence. Most HA signals were observed as punctate fluorescence in the pericentriolar region, together with diffuse fluorescence distributed throughout this region. Some SAS-6 fluorescent spots overlapped with punctate HA signals, whereas the remaining SAS-6 spots overlapped with the diffuse HA fluorescence.

In WT cells expressing HA-tagged mutant γ-tubulin [Fig. 3B; WT::γ-tub(T292I)-HA and WT::γ-tub(E89D)-HA], the overall HA localization patterns were essentially indistinguishable from those observed in the cells expressing HA-tagged WT γ-tubulin. In all cases, HA signals were detected at or around SAS-6-identified centrioles, together with both punctate and diffuse signals in the surrounding regions. Quantitative analysis showed no significant differences among the strains in the fraction of total pericentriolar HA fluorescence intensity attributable to punctate signal (Fig. 3C). Furthermore, the γ-tubulin-HA/SAS-6 fluorescence-intensity ratio, which expresses HA intensity relative to the SAS-6 intensity within the same centriole regions, did not differ significantly among the strains (Fig. 3D). Together, these results indicate that the *bld13-1* and *bld13-2* mutations do not grossly alter the subcellular localization pattern of HA-tagged γ-tubulin.

### Cytoplasmic MT organization is aberrant in *bld13* cells

In mammalian cells, γ-tubulin localizes to the PCM of the centrosome and functions in the nucleation and organization of cytoplasmic MTs (Oakley et al., 2015; Wu and Akhmanova, 2017). In *Chlamydomonas* cells, although a distinct PCM has not been observed, functional analogs of PCMs are most likely attached to the centrioles and serve as the site where γ-tubulin functions, as rootlet and non-rootlet cytoplasmic MTs extend from the proximity of the centrioles (Fig. 3A, Holmes and Dutcher, 1989). To evaluate the effects of the *bld13* mutations on the intracellular MT organization, we performed immunofluorescence microscopy using two types of tubulin antibodies. The first was an anti-acetylated tubulin antibody, which primarily recognizes axonemal and rootlet MTs (Fig. 4A), and the second was an anti-α-tubulin antibody, which recognizes both rootlet MTs and non-rootlet cytoplasmic MTs (Fig. 4B).

**Fig. 4.**
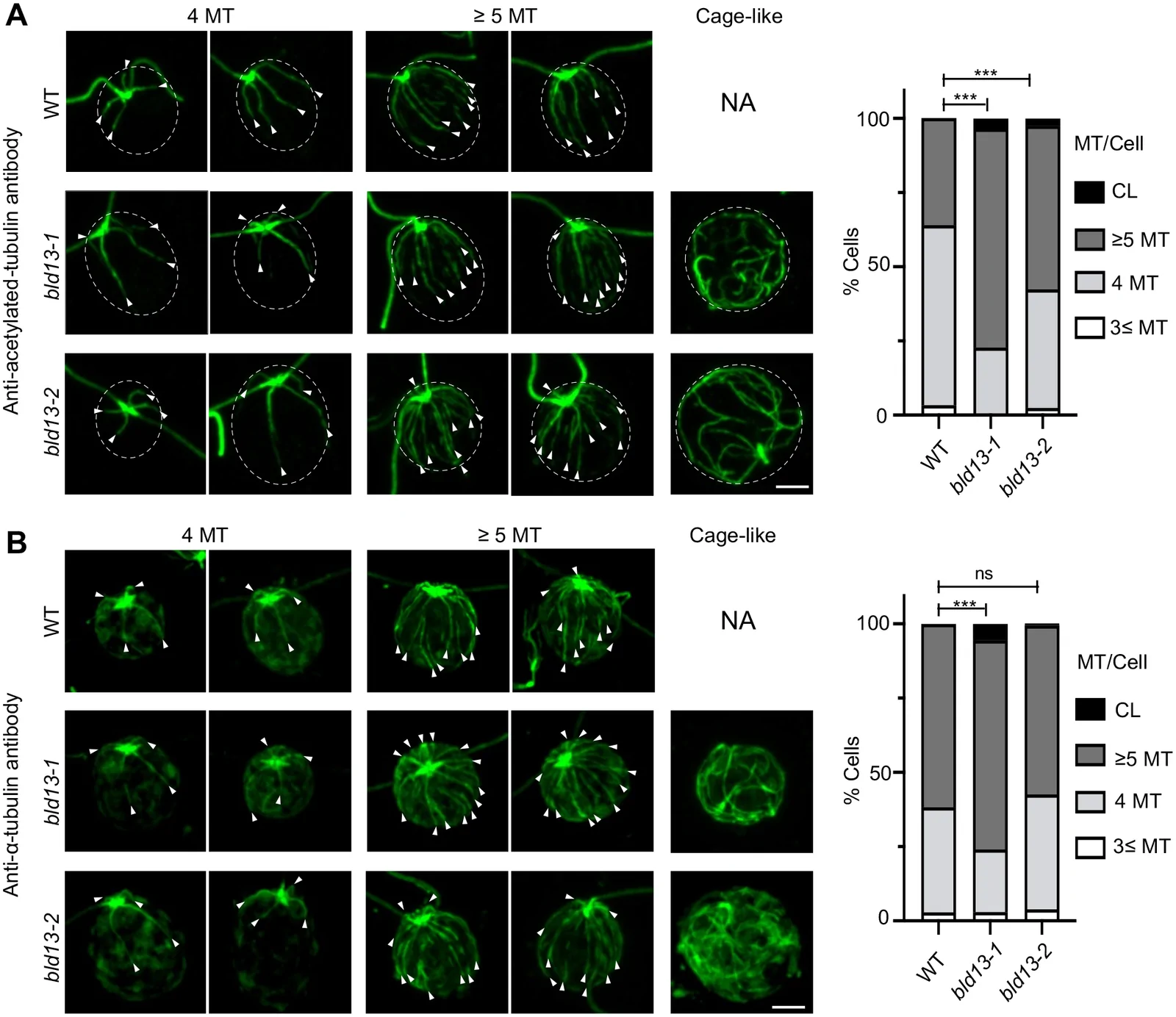
**Effects of *bld13* mutations on MT organization in *Chlamydomonas* cells.** All images are shown as Z-projection images of confocal optical sections. (A) Immunofluorescence images of WT, *bld13-1*, and *bld13-2* cells stained with anti-acetylated α-tubulin antibody, which recognizes rootlet, axonemal, and centriolar MTs, but not non-rootlet cytoplasmic MTs (Holmes and Dutcher, 1989). Cells were classified into four categories based on acetylated MT organization: cells with three or fewer acetylated MTs, cells with four MTs, cells with five or more acetylated MTs, and cells lacking an obvious MTOC but exhibiting cage-like acetylated MTs (CL). Example images of each category are shown. Arrowheads indicate rootlet or acetylated MTs with unclear rootlet identity. The graph shows the proportion of each category. (B) Immunofluorescence images of WT, *bld13-1*, and *bld13-2* cells stained with an anti-α-tubulin antibody, which labels non-rootlet cytoplasmic MTs in addition to rootlet, axonemal, and centriolar MTs. Cells were classified using the same criteria as in A, and the graph shows the proportion of cells in each category. Data are from a single experiment and therefore do not represent independent biological replicates; cell counts for each category (>120 cells per strain) are provided in Table S5. Differences in the distribution of cells across categories between each mutant and WT were assessed using Fisher's exact test on pooled cell counts (***P<0.001; ns, not significant, versus WT). Scale bar: 2 μm.

When cells were stained with the anti-acetylated α-tubulin antibody, approximately 61% of WT cells exhibited a typical cruciform rootlet arrangement of four MT bundles, consistent with the canonical rootlet organization (Fig. 4A; Table S5). In the remaining cells, five or more acetylated MT structures were observed, although their identity as rootlets could not be confirmed. In contrast, the proportion of cells with five or more acetylated MTs was higher in the mutants, reaching approximately 74% in *bld13-1* and 55% in *bld13-2*. Notably, a subset of mutant cells displayed cage-like cytoplasmic MTs that did not appear to originate from an obvious organizing center; such cells were observed at frequencies of 3.3% in *bld13-1* and 2.4% in *bld13-2* but were never observed in the WT.

Staining with the anti-α-tubulin antibody allowed visualization of non-rootlet cytoplasmic MTs in addition to rootlet MTs (Fig. 4B). Using this antibody, even WT cells frequently exhibited five or more MTs, making the distinction between WT and mutant cells less pronounced than that observed with anti-acetylated α-tubulin staining. However, in both *bld13* mutant strains, but not in WT, cells exhibiting cage-like MT arrays were again detected. In these cells, neither acetylated nor non-acetylated MTs appeared to originate from a discrete MTOC.

Taken together, these observations indicate that the *bld13-1* and *bld13-2* γ-tubulins are likely to have some defects in organizing MTs in *Chlamydomonas* cell bodies.

### Centriolar triplet MTs frequently lack protofilaments in *bld13*

The phenotypes of *bld13* mutants, namely, defective in ciliary assembly, nuclear segregation, and rootlet MT organization, are shared by various *Chlamydomonas* mutants having structural defects in the centriole (Ehler et al., 1995; Dutcher and Trabuco, 1998; Matsuura et al., 2004; Hiraki et al., 2007; Nakazawa et al., 2007). Therefore, we examined the centriole structure in *bld13* cells using thin-section electron microscopy. Longitudinal images of *bld13-1* and *bld13-2* centrioles appeared normal (Fig. 5A), although some of those centrioles did not attach axonemes and therefore apparently failed to nucleate ciliary growth. Strikingly, however, obvious defects were observed in cross-sectional images of centrioles in both mutants: some triplet MTs partially lacked protofilaments (Fig. 5B). In most cases, two to seven adjacent protofilaments were lost from particular positions in the A- and C-tubules, but not in the B-tubules. These abnormalities were rare in WT centrioles (Table 2; Tables S8 and S9). To evaluate the cross-sectional positions in the *bld13* centrioles where protofilament loss frequently occurs, the cross-sectional images of the A- or C-tubules were divided into six or 12 sectors, and the occurrence of missing protofilaments was scored in each sector. The results showed that, although the detailed sector distributions varied among sample preparations and genotypes, the protofilament loss frequently occurred in the sectors that contain protofilament nos A1–A6 of the A-tubule and nos C1–C7 of the C-tubule in both *bld13-1* and *bld13-2* (the numbering is after Linck and Stephens, 2007, and Li et al., 2019). These data indicate that protofilament loss occurred non-randomly, with a significantly biased distribution in both A- and C-tubules, whereas no protofilament loss was detected in the B-tubules (Fig. 5C; Fig. S3). A similar regional bias of protofilament loss in the A- and C-tubules was also observed in the diploid *bld13-1*/*bld13-2* strain (Fig. S3).

**Fig. 5.**
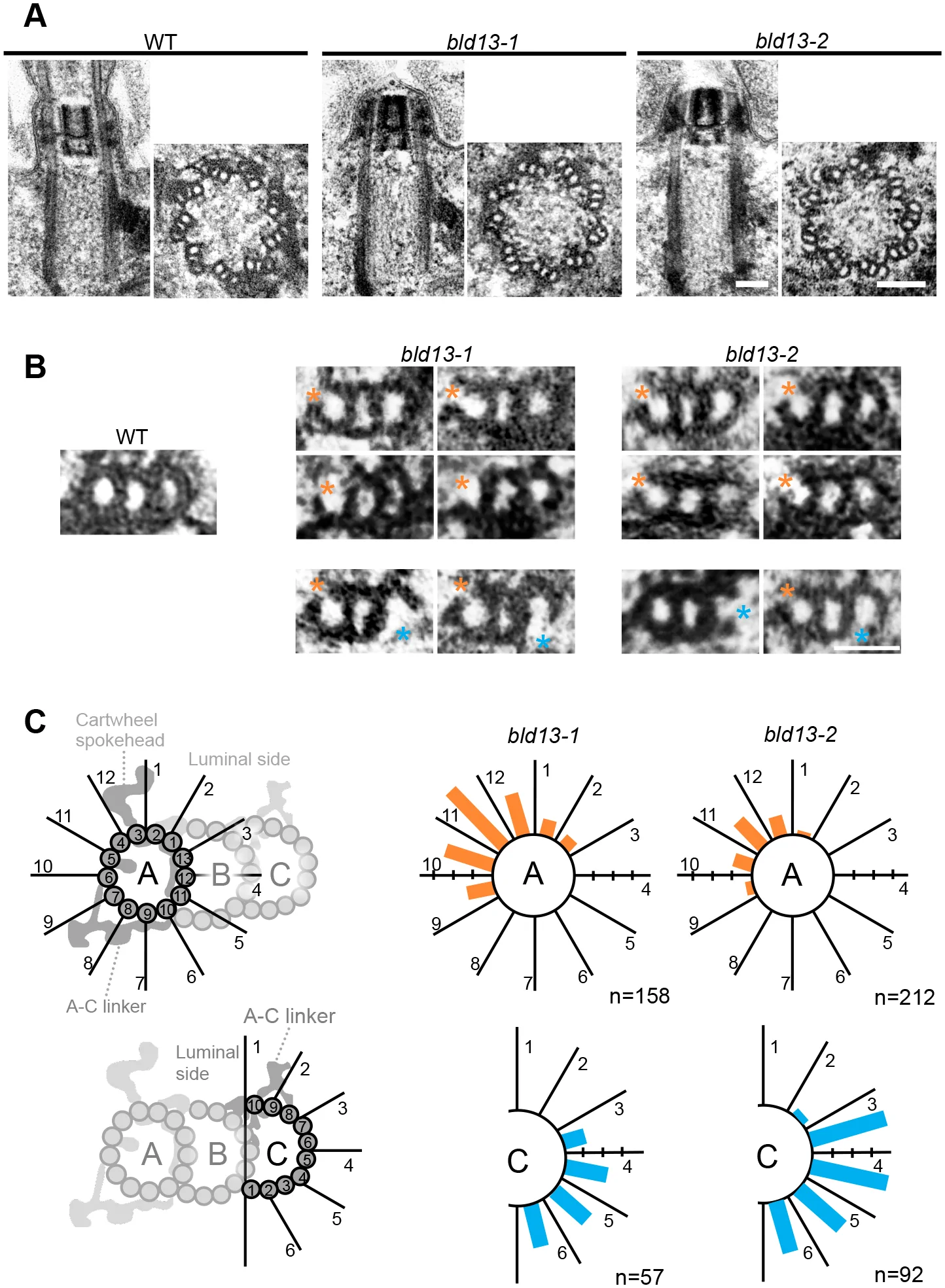
**Partial loss of protofilaments in *bld13* centriolar triplet MTs.** (A) Electron microscopic images of longitudinal and cross-sectional views of centrioles in WT, *bld13-1*, and *bld13-2* cells. Scale bars: 100 nm. (B) Magnified views of cross sections of the triplet MTs in WT, *bld13-1*, and *bld13-2* centrioles. Asterisks indicate the positions of protofilament loss. Scale bar: 40 nm. (C) Distributions of protofilament loss in A- and C-tubules of *bld13-1* and *bld13-2* triplet MTs. A cross-sectional image of the A- or C-tubule was divided into 12 or six sectors as shown in schematic diagrams (left, see Materials and Methods). MIPs (in the lumen of the A-tubule), the A–C linker, and the cartwheel spoke head are drawn in gray (Li et al., 2019). The length of each bar in the polar histograms (right) represents the percentage of protofilament loss in each sector relative to the total number of observed triplet MTs. The x-axis of each graph is shown in 5% increments. Data were obtained from a single sample preparation and represent pooled counts from multiple electron microscopy images. Results from independent biological replicates are shown in Fig. S3. Statistical analysis was performed using the Hodges–Ajne test, showing that all distributions of protofilament loss are significantly nonuniform: *bld13-1* A-tubule (*P*<0.0001; median: sector 11), *bld13-2* A-tubule (*P*<0.0001; median: sector 11), *bld13-1* C-tubule (*P*<0.0001; median: sector 4), *bld13-2* C-tubule (*P*<0.0001; median: sector 4). Raw counts underlying these distributions are provided in Table S6.

**Table 2.** Frequencies of triplet images with partial protofilament loss (%)

		Regions in longitudinal axis of the centriole (%)			
Strain		Transition zone	Distal	Central	Proximal
WT	A-tubule	ND	0/50 (0.0)	0/51 (0.0)	0/33 (0.0)
	C-tubule	ND	ND	0/50 (0.0)	ND
*bld13-1*	A-tubule	0/68 (0.0)	2/50 (4.0)	17/73 (23.3)	13/35 (37.1)
	C-tubule	ND	ND	7/57 (12.3)	ND
*bld13-2*	A-tubule	0/66 (0.0)	0/67 (0.0)	13/115 (11.3)	5/30 (16.7)
	C-tubule	ND	ND	22/92 (23.9)	ND

Notes: Percentages indicate the proportion of observed triplet MT images showing loss of one or more protofilaments. Values are given as *n*/*N*, where *n* is the number of triplets with protofilament loss and *N* is the total number of triplet MTs analyzed from electron microscopy images. Data were obtained from a single sample preparation. Corresponding values from independent biological replicates are shown in Table S8. ND, not determined.

The frequency of protofilament loss in A-tubules appeared to vary along the longitudinal axis of the centriole. To objectively assess this variation, we classified centriolar cross sections into four regions based on structural markers and quantified the occurrence of protofilament loss in each region (Table 2; Tables S8 and S9; see Materials and Methods for details). This analysis revealed that the protofilament loss of A-tubules was observed more frequently in the proximal region than in the central or distal regions. No protofilament loss was observed in the transition zone. We were unable to examine potential regional differences in C-tubules because their images were obscured by electron-dense material in the proximal region and were frequently absent in the distal region*.*

## DISCUSSION

In this study, we demonstrated that two novel mutations in the γ-tubulin gene in *Chlamydomonas* cause partial loss of protofilaments in centriolar triplet MTs while having relatively mild effects on the overall centriole structure. The ultrastructural defects observed in the mutant centrioles highlight the crucial role of γ-tubulin in maintaining the stability of triplet MTs. This study provides the first isolation and characterization of γ-tubulin mutants in *Chlamydomonas*.

### Effects of *bld13* mutations on the γ-TuRC assembly

As γ-tubulin is an essential component of the MTOC, required for fundamental cellular processes like cell division (Paz and Lüders, 2018; Sulimenko et al., 2022), loss of γ-tubulin function results in lethality in many model organisms (Sunkel et al., 1995; Hendrickson et al., 2001; Yuba-kubo et al., 2005; Pastuglia et al., 2006). Consistent with its indispensable role, data from Phytozome (https://phytozome-next.jgi.doe.gov/report/gene/CreinhardtiiCC_4532_v6_1/Cre06.g299300_4532) and the *Chlamydomonas* Library Project (https://www.chlamylibrary.org/showGene?geneIdentifier=Cre06.g299300) indicate that the γ-tubulin gene is likely essential in *Chlamydomonas* as well; high-confidence insertions in these library mutants are exclusively located in the 3′ UTR of the γ-tubulin gene and lack obvious phenotypes, suggesting that these strains still express functional WT protein. It is fortunate that the *bld13* mutants reported in this study have only mild phenotypic aberrations and thus can provide a tractable system to dissect the *in vivo* functions of γ-tubulin.

In many eukaryotes, γ-tubulin constitutes two types of complexes, one termed the γ-tubulin small complex (γ-TuSCs) and the other termed the γ-TuRC. The γ-TuSC is formed by association between γ-tubulin and two subunits, GCP2 and GCP3; the γ-TuRC is formed by oligomerization of γ-TuSCs and association with GCP4∼6 and MZT1 (Tovey and Conduit, 2018; Böhler et al., 2021). Because *Chlamydomonas* has genes coding for homologs of GCP2∼4 and MZT1, γ-tubulin of this organism most likely also functions in MT nucleation by forming γ-TuRCs.

Both *bld13* mutations in the γ-tubulin gene cause single-amino-acid substitutions without altering the charge of the side chain, although T292I represents a less conservative substitution than E89D (Fig. 1D). Consistently, AlphaFold2 predicts no significant changes in the overall molecular structure (Fig. S1A). The predicted minor structural effects of these mutations may be correlated with the mild phenotypic abnormalities in the mutants: the mutant γ-tubulins localize to both centrioles and the surrounding pericentriolar region as in WT cells (Silflow et al., 1999), and cytoplasmic MTs and ciliary axonemes are formed in most of the mutant cells (Figs 1–3). On closer inspection, however, the *bld13* mutations were found to be associated with three detectable abnormalities in MT structures in a subset of cells. One abnormality was an increased population of cells exhibiting elevated numbers of cytoplasmic MTs, most clearly detected by staining for acetylated α-tubulin (Fig. 4). A second abnormality, observed in a small fraction of cells, was the appearance of cytoplasmic MTs that lacked an obvious MTOC (Fig. 4). A third abnormality was a partial loss of protofilaments in centriolar MTs (Fig. 5).

We speculate that these defects could result from some instability in the association of the γ-tubulin molecules within the γ-TuRC. This is because the mutated amino acid residues in both *bld13* mutants are located on the molecular surface in the region where adjacent γ-tubulin molecules are presumed to interact within the complex (Fig. S1B,C; Inclán and Nogales, 2001; Aldaz et al., 2005; Wieczorek et al., 2020). In addition, AlphaMissense predicts that the equivalent substitutions in human γ-tubulin are ‘likely pathogenic’ (Cheng et al., 2023), further supporting the functional importance of these residues. The more pronounced phenotype of *bld13-1* than that of *bld13-2* may reflect the different biochemical consequences of the two amino acid substitutions. The T292I substitution in *bld13-1* is nonconservative, replacing a polar residue with a hydrophobic residue, whereas the E89D substitution in *bld13-2* is conservative and retains the acidic nature of the residue. Consistent with this possibility, the AlphaMissense score was higher for T288I in human γ-tubulin (corresponding to *Chlamydomonas* T292I; 0.9956) than for E89D (corresponding to *Chlamydomonas* E89D; 0.9508) (Cheng et al., 2023). Thus, T292I may perturb γ-tubulin structure or intermolecular interactions more strongly than E89D. We propose that γ-tubulin molecules are stably held in the complex by inter-subunit interaction at the contact sites, and the *bld13* mutations partially disrupt this interaction.

Diploid analyses further support the dominant-negative mode of the *bld13* mutations, which is consistent with the results of the γ-tubulin expression experiments (Fig. 2). The presence of both WT and mutant γ-tubulin in heterozygous diploids suggests that mutant subunits are incorporated into the γ-TuRC together with WT subunits, thereby reducing the overall functional integrity of the complex. Moreover, the slightly more severe phenotype observed in the *bld13-1*/*bld13-2* compound heterozygote diploid suggests that the coexistence of the two mutations may further impair inter-subunit contact surfaces within the γ-TuRC. We speculate that weakened contact between γ-tubulins may compromise γ-TuRC stability, leading to the defective cellular MT organization and protofilament loss observed in *bld13* mutants. In line with this model, the *bld13-1/bld13-2* diploid also displayed partial protofilament loss in centriolar triplet MTs, resembling that observed in the haploid *bld13* mutants (Fig. S3, Tables S7 and S9).

It remains unclear how the structural defects in the *bld13* γ-TuRC result in the abnormal organization of the rootlet and non-rootlet MTs (Fig. 4). Because the other centriole-deficient mutants we studied previously, *bld10* and *bld12*, also exhibited abnormal rootlet microtubule organization (Matsuura et al., 2004; Nakazawa et al., 2007), microtubule organization in *Chlamydomonas* may be highly susceptible to structural abnormalities of the centriole.

### γ-Tubulin functions in stabilization of centriolar MTs

The triplet MTs of *bld13* centrioles frequently lack part of the protofilaments (Fig. 5). This type of MT defect is unexpected because the lateral interaction between protofilaments is likely essential for the stability of MTs, and the loss of a protofilament would lead to depolymerization of whole MTs (Gudimchuk and McIntosh, 2021). However, the triplet MTs in *bld13*, even when lacking protofilaments, do not fully depolymerize. We speculate that these MTs are stabilized by some MT-associated structures or proteins, such as the A–C linkers, MT inner proteins (MIPs), and the inner scaffolds of the centriole (Li et al., 2019; LeGuennec et al., 2020). It is interesting to note that the protofilament loss is frequently observed in regions A1–A6 and regions C1–C7, where protofilaments are apparently not associated with those structures or MIPs (Fig. 5C; Table S6). This regional bias was consistently observed in two independently prepared samples for each of *bld13-1* and *bld13-2*, although the frequency of protofilament loss varied between sample preparations. A similar regional bias was consistently observed in two independently prepared samples of the *bld13-1/bld13-2* diploid (Fig. S3, Table S7).

Protofilament loss in A-tubules was observed more frequently in the proximal region than in the central or distal regions of *bld13-1*, *bld13-2*, and *bld13-1/bld13-2* diploid centrioles (Table 2; Tables S8 and S9), suggesting that the loss was caused by depolymerization from the proximal end of previously formed protofilaments. *In vitro* studies have shown that the γ-TuRC caps the MT minus end for a short period and suppresses both polymerization and depolymerization (Wiese and Zheng, 2000; Berman et al., 2023). In centriolar triplet MTs *in vivo*, their minus ends may be mostly capped by the γ-TuRC, since γ-tubulin has been localized near the proximal ends of the mature centrioles in *Tetrahymena*, *Paramecium*, and mammals (Fuller et al., 1995; Klotz et al., 2003; Joachimiak et al., 2018). Moreover, cryo-electron tomography of human procentrioles has shown that the A-tubule proximal ends have a conical shape resembling the γ-TuRC-capped MT end observed *in vitro* (Guichard et al., 2010). Thus, our observations support the possibility that the γ-TuRC caps the proximal end of the A-tubule to suppress depolymerization and that this capping function may be compromised in the *bld13* centrioles due to a weakened interaction between γ-tubulin molecules.

Electron microscopy of the *bld13* centrioles revealed partial loss of protofilaments in C-tubules as well (Fig. 5B,C; Fig. S3). The mechanism underlying formation and maintenance of B- and C-tubules in centrioles remains poorly understood, although several proteins, including δ-tubulin, ε-tubulin, and CPAP/SAS-4, have been associated with these processes (Hung et al., 2000; Dutcher, 2003; Gogendeau et al., 2011; Zheng et al., 2016; Stathatos et al., 2021; Vásquez-Limeta et al., 2022; Guichard et al., 2023). Unlike the A-tubule, which appears to have a capped proximal end, the B- and C-tubules have open proximal ends, suggesting that their formation and stabilization rely on mechanisms distinct from those of the A-tubule. Interestingly, however, a previous study reported that overexpression of a γ-tubulin variant in *Tetrahymena* resulted in the loss of B- and C-tubules in basal bodies (Joachimiak et al., 2018), raising the possibility that γ-tubulin also contributes to the assembly or stability of these tubules. Consistent with this idea, we observed protofilament loss from the centriolar C-tubule in the γ-tubulin mutants. One possibility is that γ-TuRCs localized to the outer wall of the centriole contribute to the stabilization of the C-tubule and possibly the B-tubule as well (Schweizer and Lüders, 2021; Kalbfuss and Gönczy, 2023).

In summary, we found that the *bld13* mutations in γ-tubulin lead to a partial loss of protofilaments in centriolar triplet MTs, while the triplet structure remains stable even when some protofilaments are lost. The loss of protofilaments appears to proceed from the proximal end, potentially due to reduced minus-end capping by the γ-TuRC containing *bld13* γ-tubulin. Our findings have thus highlighted a potential role for γ-tubulin and the γ-TuRC in the stabilization of the minus end of the centriole MTs, in addition to their established function in initiating tubulin polymerization. Future structural analyses of γ-TuRCs containing *bld13* γ-tubulin could first examine whether these mutations cause local alterations in the γ-tubulin array and, if so, could then provide insight into how such alterations might affect γ-TuRC organization and function. Such structural analyses could be complemented by functional analyses of mild mutations in other γ-TuRC components and centriolar factors, which would help assess whether partial perturbation of γ-TuRC function produces comparable defects in centriolar triplet MTs. Together, these structural and functional approaches could help clarify how γ-TuRC contributes to the stabilization of centriolar triplet MTs.

## MATERIALS AND METHODS

### *C. reinhardtii* strains

The WT strains CC124, CC125, and S1-D2 were obtained from the *Chlamydomonas* Resource Center (University of Minnesota). The mutant strains *bld13-1* and *bld13-2* were isolated in this study by screening CC125 cells treated with sodium azide or irradiated with ultraviolet (UV), respectively. The isolated strains were then backcrossed twice with WT, and the resulting F2 progeny were used for all subsequent analyses. The diploid strains (WT/WT, WT/*bld13-1*, WT/*bld13-2*, *bld13-1*/*bld13-2*) were produced by crossing two strains with different antibiotic resistance markers: hygromycin and paromomycin resistance, each conferred by transformation with a plasmid pHyg3 carrying the *aphVII* gene or pSI103 carrying the *aphVIII* gene using electroporation (Ebersold, 1967; Berthold et al., 2002; Sizova et al., 2001; Yamano et al., 2013). The cells were grown in Tris-acetate-phosphate (TAP) medium under constant illumination (Gorman and Levine, 1965).

### Mutagenesis and screening centriole mutants

Random mutagenesis was performed by UV irradiation or sodium azide treatment. For UV mutagenesis, approximately 1000 cells were spread on 90 mm TAP agar plates and irradiated with UV light for 30–45 s, based on previously described procedures for *Chlamydomonas* UV mutagenesis (Lewin, 1952), with modifications. The plates were incubated in the dark overnight and then cultured under continuous light for 7 days. For chemical mutagenesis, sodium azide treatment was performed using a procedure modified from the N-methyl-N′-nitro-N-nitrosoguanidine (MNNG) mutagenesis method for *C. reinhardtii* (Huang et al., 1977), with sodium azide used as the mutagen based on its established mutagenicity (Kleinhofs et al., 1978). Cells were suspended in 0.1 M citrate buffer (pH 5.0) containing 0.25 mM sodium azide and incubated in the dark for 60 min. The cells were then washed, plated on TAP agar plates, and incubated under continuous light for 7 days.

The colonies obtained after mutagenesis were individually picked and transferred to a 96-well plate containing liquid TAP medium. The clones were cultured for 2–3 days and then screened by light microscopy for motility defects. Clones containing nonmotile cells or cells exhibiting partially abnormal motility, such as spinning, were selected as candidate mutants. For these clones, the numbers of cilia and nuclei per cell were determined to identify mutants potentially defective in centriole assembly or function, as described below.

### Cilia and nuclear counts

For cilia counting, the cells were treated with autolysin to remove the cell wall, and ciliary motility was then inhibited by treating the cells with 0.1 mM NiCl_2_, essentially as described previously (Bean and Harris, 1979). Immobilized cells were observed using phase-contrast microscopy (Axioplan, Zeiss), and the number of cilia per cell was determined by visual inspection. For nuclear counting, the cells were similarly treated with autolysin, and the DNA was stained with SYBR Green I (Sigma-Aldrich), following established protocols as described previously (Nishimura et al., 1998). The stained cells were observed using fluorescence microscopy (Axioplan, Zeiss), and the number of nuclei per cell was determined by visual inspection.

### Expression of HA-tagged γ-tubulin in *Chlamydomonas*

Genome fragments containing the γ-tubulin gene (Cre06.g299300, https://phytozome-next.jgi.doe.gov/) were amplified by PCR with the primers γ-tub-F (5′-AACTCCCTCACCACCCAGTCA-3′) and γ-tub-R (5′-TTTCATTAGGGACCAGGTTAG-3′) using genomic DNAs of the WT, *bld13-1* or *bld13-2*. The forward primer (γ-tub-F) was designed to amplify approximately 500 bp upstream of the 5′ UTR of the γ-tubulin gene, thereby including its putative native promoter region within the amplified fragment. The amplified fragments were cloned into a pCR-Blunt II-TOPO vector (Invitrogen). Into this plasmid, a *Spe*I fragment containing the *aphVII* gene excised from pHyg3 was inserted at the *Spe*I site, and a fragment containing 3×HA-tag sequences digested with *Nru*I/*Sca*I from p3×HA (Silflow et al., 2001) was inserted at the site adjacent to the stop codon of the γ-tubulin gene. These plasmids were introduced into the WT, *bld13-1* or *bld13-2* cells by electroporation using the NEPA21 electroporator (Nepa Gene, Japan) (Yamano et al., 2013). Of the transformants that display resistance to hygromycin, the clones that express the HA-tagged γ-tubulin were screened by western blotting using an anti-HA antibody (3F10, Roche). For each combination of strain background and HA-tagged γ-tubulin construct, a single transformant showing comparable levels of HA-tagged γ-tubulin expression was selected. The selected transformants, designated according to their strain background and γ-tubulin construct, were used throughout the experiments presented in Figs 2 and 3.

### Genetic mapping and whole-genome sequencing

The mutants *bld13-1* and *bld13-2* were mapped to a region containing the γ-tubulin gene on Chromosome 6 using amplified fragment length polymorphism (AFLP)-based analysis of the progeny resulting from crossing these mutants with S1-D2 (Kathir et al., 2003; Nakazawa et al., 2007). Recombination frequencies between each mutation and genetic markers on Chromosome 6 were determined by detecting polymorphisms in PCR products. The genetic markers used were as follows: 19-559 (5′-CAGTGTGTAGGGTTGAGCACAGGA-3′, 5′-CAGCCGGAGATGTTTACGTTTGAG-3′), 190065 (5′-GGGGCACTGACGTCAGGCAAG-3′, 5′-GCAAAGCGTCTAGCCTGGACGAA-3′, 5′-GGATACTGGACTACTTTCACGCTGCA-3′), 190071 (5′-GGCCCAGTCGATGCTGGTGTTCGG-3′, 5′-GTCTGGGTCGGCGCCCTTGAGA-3′, 5′-GGGGCACACATGATGAGAGGTGCG-3′), TUG (5′-GTCGCCAGGAATTTTGCCCCTGG-3′, 5′-GCGCGCCTGGCGGTAGCACATA-3′, 5′-AGCAGCGCTATGTTCGCTTCCCC-3′), 19-1050 (5′-AGGCAGGGGAGACATAGGTAGGAG-3′, 5′-GACCCTATGACCACCATCTGTTCC-3′), and 9-0003 (5′-GTAGCACCATATGGGTGGAGTGG-3′, 5′-AGAACTGCGCCAAGATTTGCACG-3′). Of these markers, no recombinants were detected between the mutations and markers 190071, TUG, and 19-1050 among the 188 mutant progeny examined.

Whole-genome sequencing was performed using genomic DNA prepared from *bld13-1* cells pretreated with gametolysin to remove the cell wall (Kindle, 1990) using the DNeasy Plant Mini kit (Qiagen). A total of 100 ng of genomic DNA was fragmented to an average size of ∼350 bp using a Covaris sonicator. Sequence libraries were constructed from the DNA fragments using the TruSeq Nano DNA Library Prep Kit (Illumina), essentially following the manufacturer's instructions. To minimize PCR amplification bias, the number of PCR cycles in the amplification enrichment step was reduced to four. The generated library was evaluated using the Bioanalyzer (Agilent) and then sequenced on a HiSeq 2500 (Illumina) in a 101×101 paired-end format.

The sequence reads were aligned onto the *Chlamydomonas* genome sequence version 5.3.1 (Creinhardtii_236.fa.gz) downloaded from the Joint Genome Institute (JGI) (https://phytozome.jgi.doe.gov/pz/portal) using bowtie2 (bowtie-bio.sourceforge.net/bowtie2/index.shtml). The *bld13-1* mutation was identified by comparing the genome sequence of *bld13-1* with that of CC124 and CC503, focusing on the mapped ∼400 kb region on Chromosome 6. The *bld13-2* mutation was identified through PCR amplification and sequencing of the γ-tubulin gene in the *bld13-2* genome.

### Western blot analysis

For immunoblotting, whole-cell extracts (10 μg per lane) from *Chlamydomonas* cells were separated by SDS-PAGE and transferred to Immobilon-P membranes (Millipore) as described by Noga et al. (2022). Anti-HA antibody bound to the membrane was detected using a goat anti-rat IgG conjugated with horseradish peroxidase (HRP) (Sigma-Aldrich).

### Immunofluorescence microscopy

*Chlamydomonas* cells were fixed and prepared for antibody staining as described previously (Arakaki et al., 2017). Anti-HA antibody (3F10, Roche), anti-α-tubulin antibody (B-5-1-2, Sigma-Aldrich), rabbit anti-SAS-6 antibody (Nakazawa et al., 2007), and anti-acetylated α-tubulin antibody (6-11B-1, Sigma-Aldrich) were used as primary antibodies. Goat anti-mouse IgG conjugated with Alexa Fluor 647 (Thermo Fisher Scientific), goat anti-mouse IgG(H+L) cross-adsorbed secondary antibody conjugated with Alexa Fluor 488 (Thermo Fisher Scientific), goat anti-rat IgG conjugated with Alexa Fluor 488 (Thermo Fisher Scientific), and goat anti-rabbit IgG conjugated with Alexa Fluor 546 (Thermo Fisher Scientific) were used as secondary antibodies. Images were acquired using a Nikon AX confocal microscope equipped with an NSPARC super-resolution detector (Nikon Instruments Inc.). For the observation of cytoplasmic MTs, Z-stacks were collected at 0.15 μm intervals, and Z-projected images were generated using maximum-intensity projection of 40–50 optical sections.

### Quantification of punctate HA-tagged γ-tubulin intensity in the pericentriolar region

To quantify punctate γ-tubulin-HA signal around centrioles for Fig. 3C, a fixed-size elliptical region of interest (ROI; approximately 1.38×1.25 μm; 1.35 μm^2^) enclosing the pericentriolar region was defined using the HA channel in ImageJ and saved in the ROI Manager. The same ROI was then positioned over the pericentriolar region of each centriole pair. Within this ROI, integrated γ-tubulin-HA fluorescence intensity was measured as the total pericentriolar signal. Within the same ROI, punctate γ-tubulin-HA signals were identified in the HA channel by adjusting the intensity threshold for each cell to account for cell-to-cell variation in overall fluorescence intensity. Threshold adjustment was performed using the same visual criterion: correspondence of the threshold overlay to visible punctate γ-tubulin-HA structures. The integrated fluorescence intensity of the thresholded puncta was measured as the punctate signal.

For each cell, background mean fluorescence intensity was measured in two fixed-size elliptical HA-negative regions (approximately 0.95×0.60 μm; 0.45 μm^2^) and averaged. Background fluorescence was subtracted from both the total pericentriolar and punctate signals by multiplying the mean background intensity by the area of the corresponding measurement region. The resulting background-subtracted values were then used to calculate the puncta/total pericentriolar intensity ratio for each cell. Cells were excluded if punctate γ-tubulin-HA structures could not be reliably identified using the above-described thresholding criterion. Approximately 20 cells per strain expressing γ-tubulin-HA were analyzed, and the exact numbers are indicated in the figure legend. All measurements were performed blind to genotype. The ratios were plotted as individual data points with mean±s.d., and multiple comparisons among strains were performed using a one-way analysis of variance (ANOVA) followed by a Dunnett's test in GraphPad Prism.

### Quantification of γ-tubulin-HA at SAS-6-positive centrioles

To quantify the association between γ-tubulin-HA and SAS-6 at centrioles for Fig. 3D, SAS-6-positive signals corresponding to the mature centriole pair were identified in the SAS-6 channel. A fixed-size circular ROI (approximately 200 nm in diameter) was centered on each SAS-6-positive signal. The ROI size was selected to provide a consistent measurement window while minimizing overlap between the two closely positioned centrioles. Cells in which the two SAS-6-positive signals corresponding to the mature centriole pair were not sufficiently resolved for reliable placement of separate ROIs were excluded. The integrated fluorescence intensities of SAS-6 and γ-tubulin-HA were measured within each ROI, and background subtraction was performed as described above for Fig. 3C. The background-subtracted intensities from the two mature centrioles were averaged separately for SAS-6 and γ-tubulin-HA to obtain one value per channel per cell. The γ-tubulin-HA/SAS-6 fluorescence intensity ratio was then calculated for each cell to express the HA intensity relative to the SAS-6 intensity within the same centriole regions. Approximately 20 cells per strain expressing γ-tubulin-HA were analyzed, and the exact numbers are indicated in the figure legend. All measurements were performed blind to genotype. The ratios were plotted as individual data points with mean±s.d., and the differences between WT::γ-tub(WT)-HA and each of the other strains were assessed using a one-way ANOVA followed by a Dunnett's test in GraphPad Prism.

### Quantification of MT defects

For quantification of MT organization (Fig. 4), *Z*-projected immunofluorescence images of cells stained with anti-acetylated α-tubulin antibody (Fig. 4A) or anti-α-tubulin antibody (Fig. 4B) were analyzed. The cells were categorized into four groups based on the number and organization of the stained MTs: cells with three or fewer MTs, cells with four MTs, cells with five or more MTs, and cells with cage-like MTs that did not originate from an obvious organizing center (CL). Cells were excluded when the staining quality was insufficient to allow reliable classification of MT number and organization. For each strain, more than 120 cells were randomly selected and manually scored. Statistical analysis was performed using Fisher's exact test in GraphPad Prism to compare the distribution of MT categories between WT and mutant strains. All scoring was performed blind to genotype. The statistical power of 0.8 or higher was confirmed for each comparison in a post hoc manner using G*Power (Faul et al., 2007).

### Electron microscopy

Logarithmically growing cells were prefixed with 2% glutaraldehyde in 0.1 M S-collidine buffer for 1–2 h at 0°C and postfixed with 1% OsO_4_ in the same buffer solution for 1–2 h (Wright et al., 1983). The specimens were then dehydrated and embedded in EPON 812. Thin sections (50–70 nm) were stained with aqueous uranyl acetate and Reynolds' lead citrate. The primary EM dataset underlying Fig. 5, Table 2 and Table S6 was acquired using a JEM-1400Plus (JEOL) equipped with an EM-14800RUBY digital CCD camera (JEOL) and a JEM-1230R (JEOL) equipped with an UltraScan 1000 CCD camera (model 894; Gatan). The independent replicate dataset underlying Fig. S3 and Tables S7–S9 was acquired using a JEM-1400Flash (JEOL) equipped with a Matataki Flash high-sensitivity sCMOS camera (JEOL). The replicate dataset comprised two separately prepared samples acquired under the same imaging conditions and pooled for analysis.

### Analysis of protofilament loss in centriolar triplet MTs

To evaluate the integrity of triplet MT, we collected electron micrographs of A-tubules from the distal, central and proximal regions, as well as C-tubules from the central region, of *bld13-1*, *bld13-2*, and *bld13-1/bld13-2* centrioles, selecting images in which the protofilament arrangement was sufficiently clear for analysis. Potential intrinsic variation among the nine triplets within each centriole was not considered during image selection. Each tubule image was divided into sectors (12 for the A-tubule and six for the C-tubule), and the number of missing protofilaments in each sector was scored (Fig. 5C; Fig. S3). Sectors rather than numbered protofilaments were used because the images often lacked sufficient resolution to identify specific protofilament numbers. No protofilament loss was observed in B-tubules.

For the analysis in Table 2, S8 and S9, the longitudinal position of each centriole cross section was determined from characteristic ultrastructural landmarks, following previous descriptions of centriole architecture (Geimer and Melkonian, 2004; LeGuennec et al., 2021). Cross sections were classified into four regions: the transition zone, identified by the characteristic starlike luminal structure; the distal region, identified by the presence of distal appendages; the proximal region, identified by the presence of the cartwheel; and the central region, identified by the presence of triplet MTs and the absence of the diagnostic structures of the other three regions. For each region, the percentages of A- and C-tubules showing protofilament loss were calculated.

To assess whether protofilament loss was uniformly distributed among sectors or showed a preferential orientation, circular statistics were employed. The Hodges–Ajne test was performed for each genotype using a custom MATLAB script. Sample sizes were initially determined based on experimental feasibility, and a statistical power of 0.8 or higher was confirmed post hoc for each comparison using G*Power (Faul et al., 2007).

## Supplementary Material

10.1242/biolopen.062609_sup1 Supplementary information

## References

[BIO062609C1] Aldaz, H., Rice, L. M., Stearns, T. and Agard, D. A. (2005). Insights into microtubule nucleation from the crystal structure of human gamma-tubulin. *Nature* 435, 523-527. 10.1038/nature03586 15917813

[BIO062609C2] Arakaki, Y., Fujiwara, T., Kawai-Toyooka, H., Kawafune, K., Featherston, J., Durand, P. M., Miyagishima, S. Y. and Nozaki, H. (2017). Evolution of cytokinesis-related protein localization during the emergence of multicellularity in volvocine green algae. *BMC Evol. Biol.* 17, 243. 10.1186/s12862-017-1091-z PMC5717801 29212441

[BIO062609C3] Azimzadeh, J. and Marshall, W. F. (2010). Building the centriole. *Curr. Biol.* 20, R816-R825. 10.1016/j.cub.2010.08.010 PMC2956124 20869612

[BIO062609C4] Bahtz, R., Seidler, J., Arnold, M., Haselmann-Weiss, U., Antony, C., Lehmann, W. D. and Hoffmann, I. (2012). GCP6 is a substrate of Plk4 and required for centriole duplication. *J. Cell Sci.* 125, 486-496. 10.1242/jcs.093930 22302995

[BIO062609C5] Bean, B. and Harris, A. (1979). Selective inhibition of flagellar activity in *Chlamydomonas* by nickel. *J. Protozool.* 26, 235-240. 10.1111/j.1550-7408.1979.tb02769.x 114631

[BIO062609C6] Berman, A. Y., Wieczorek, M., Aher, A., Olinares, P. D. B., Chait, B. T. and Kapoor, T. M. (2023). A nucleotide binding-independent role for γ-tubulin in microtubule capping and cell division. *J. Cell Biol.* 222, e202204102. 10.1083/jcb.202204102 PMC9930161 36695784

[BIO062609C7] Berthold, P., Schmitt, R. and Mages, W. (2002). An engineered *Streptomyces hygroscopicus* aph 7″ gene mediates dominant resistance against hygromycin B in *Chlamydomonas reinhardtii*. *Protist* 153, 401-412. 10.1078/14344610260450136 12627869

[BIO062609C8] Böhler, A., Vermeulen, B. J. A., Würtz, M., Zupa, E., Pfeffer, S. and Schiebel, E. (2021). The gamma-tubulin ring complex: Deciphering the molecular organization and assembly mechanism of a major vertebrate microtubule nucleator. *BioEssays* 43, e2100114. 10.1002/bies.202100114 34160844

[BIO062609C9] Cheng, J., Novati, G., Pan, J., Bycroft, C., Žemgulytė, A., Applebaum, T., Pritzel, A., Wong, L. H., Zielinski, M., Sargeant, T. et al. (2023). Accurate proteome-wide missense variant effect prediction with AlphaMissense. *Science* 381, eadg7492. 10.1126/science.adg7492 37733863

[BIO062609C10] Cota, R. R., Teixidó-Travesa, N., Ezquerra, A., Eibes, S., Lacasa, C., Roig, J. and Lüders, J. (2017). MZT1 regulates microtubule nucleation by linking γTuRC assembly to adapter-mediated targeting and activation. *J. Cell Sci.* 130, 406-419. 10.1242/jcs.195321 27852835

[BIO062609C11] Dammermann, A., Müller-Reichert, T., Pelletier, L., Habermann, B., Desai, A. and Oegema, K. (2004). Centriole assembly requires both centriolar and pericentriolar material proteins. *Dev. Cell* 7, 815-829. 10.1016/j.devcel.2004.10.015 15572125

[BIO062609C12] Dutcher, S. K. (2003). Long-lost relatives reappear: identification of new members of the tubulin superfamily. *Curr. Opin. Microbiol.* 6, 634-640. 10.1016/j.mib.2003.10.016 14662361

[BIO062609C13] Dutcher, S. K. and Trabuco, E. C. (1998). The UNI3 gene is required for assembly of basal bodies of *Chlamydomonas* and encodes delta-tubulin, a new member of the tubulin superfamily. *Mol. Biol. Cell* 9, 1293-1308. 10.1091/mbc.9.6.1293 PMC25351 9614175

[BIO062609C14] Ebersold, W. T. (1967). *Chlamydomonas reinhardi*: heterozygous diploid strains. *Science* 157, 447-449. 10.1126/science.157.3787.447 6028032

[BIO062609C15] Ehler, L. L., Holmes, J. A. and Dutcher, S. K. (1995). Loss of spatial control of the mitotic spindle apparatus in a *Chlamydomonas reinhardtii* mutant strain lacking basal bodies. *Genetics* 141, 945-960. 10.1093/genetics/141.3.945 PMC1206857 8582639

[BIO062609C16] Faul, F., Erdfelder, E., Lang, A. G. and Buchner, A. (2007). G*Power 3: a flexible statistical power analysis program for the social, behavioral, and biomedical sciences. *Behav. Res. Methods* 39, 175-191. 10.3758/BF03193146 17695343

[BIO062609C17] Fuller, S. D., Gowen, B. E., Reinsch, S., Sawyer, A., Buendia, B., Wepf, R. and Karsenti, E. (1995). The core of the mammalian centriole contains gamma-tubulin. *Curr. Biol.* 5, 1384-1393. 10.1016/S0960-9822(95)00276-4 8749391

[BIO062609C18] Geimer, S. and Melkonian, M. (2004). The ultrastructure of the *Chlamydomonas reinhardtii* basal apparatus: identification of an early marker of radial asymmetry inherent in the basal body. *J. Cell Sci.* 117, 2663-2674. 10.1242/jcs.01120 15138287

[BIO062609C19] Gogendeau, D., Hurbain, I., Raposo, G., Cohen, J., Koll, F. and Basto, R. (2011). Sas-4 proteins are required during basal body duplication in *Paramecium*. *Mol. Biol. Cell.* 22, 1035-1044. 10.1091/mbc.e10-11-0901 PMC3069007 21289083

[BIO062609C20] Gorman, D. S. and Levine, R. P. (1965). Cytochrome f and plastocyanin: their sequence in the photosynthetic electron transport chain of *Chlamydomonas reinhardi*. *Proc. Natl. Acad. Sci. USA* 54, 1665-1669. 10.1073/pnas.54.6.1665 PMC300531 4379719

[BIO062609C21] Gudimchuk, N. B. and McIntosh, J. R. (2021). Regulation of microtubule dynamics, mechanics and function through the growing tip. *Nat. Rev. Mol. Cell Biol.* 22, 777-795. 10.1038/s41580-021-00399-x 34408299

[BIO062609C22] Guichard, P., Chrétien, D., Marco, S. and Tassin, A. M. (2010). Procentriole assembly revealed by cryo-electron tomography. *EMBO J.* 29, 1565-1572. 10.1038/emboj.2010.45 PMC2876950 20339347

[BIO062609C23] Guichard, P., Laporte, M. H. and Hamel, V. (2023). The centriolar tubulin code. *Semin. Cell Dev. Biol.* 137, 16-25. 10.1016/j.semcdb.2021.12.001 34896019

[BIO062609C24] Hendrickson, T. W., Yao, J., Bhadury, S., Corbett, A. H. and Joshi, H. C. (2001). Conditional mutations in gamma-tubulin reveal its involvement in chromosome segregation and cytokinesis. *Mol. Biol. Cell.* 12, 2469-2481. 10.1091/mbc.12.8.2469 PMC58607 11514629

[BIO062609C25] Hiraki, M., Nakazawa, Y., Kamiya, R. and Hirono, M. (2007). Bld10p constitutes the cartwheel-spoke tip and stabilizes the 9-fold symmetry of the centriole. *Curr. Biol.* 17, 1778-1783. 10.1016/j.cub.2007.09.021 17900905

[BIO062609C26] Holmes, J. A. and Dutcher, S. K. (1989). Cellular asymmetry in *Chlamydomonas reinhardtii**.* *J. Cell Sci.* 94, 273-285. 10.1242/jcs.94.2.273 2621224

[BIO062609C27] Huang, B., Rifkin, M. R. and Luck, D. J. (1977). Temperature-sensitive mutations affecting flagellar assembly and function in *Chlamydomonas reinhardtii*. *J. Cell Biol.* 72, 67-85. 10.1083/jcb.72.1.67 PMC2110986 830657

[BIO062609C28] Hung, L. Y., Tang, C. J. and Tang, T. K. (2000). Protein 4.1 R-135 interacts with a novel centrosomal protein (CPAP) which is associated with the gamma-tubulin complex. *Mol. Cell Biol.* 20, 7813-7825. 10.1128/MCB.20.20.7813-7825.2000 PMC86375 11003675

[BIO062609C29] Inclán, Y. F. and Nogales, E. (2001). Structural models for the self-assembly and microtubule interactions of gamma-, delta- and epsilon-tubulin. *J. Cell Sci.* 114, 413-422. 10.1242/jcs.114.2.413 11148142

[BIO062609C30] Joachimiak, E., Jerka-Dziadosz, M., Krzemień-Ojak, Ł., Wacławek, E., Jedynak, K., Urbanska, P., Brutkowski, W., Sas-Nowosielska, H., Fabczak, H., Gaertig, J. et al. (2018). Multiple phosphorylation sites on γ-tubulin are essential and contribute to the biogenesis of basal bodies in Tetrahymena. *J. Cell. Physiol.* 233, 8648-8665. 10.1002/jcp.26742 29761930

[BIO062609C31] Jumper, J., Evans, R., Pritzel, A., Green, T., Figurnov, M., Ronneberger, O., Tunyasuvunakool, K., Bates, R., Žídek, A., Potapenko, A. et al. (2021). Highly accurate protein structure prediction with AlphaFold. *Nature* 596, 583-589. 10.1038/s41586-021-03819-2 PMC8371605 34265844

[BIO062609C32] Kalbfuss, N. and Gönczy, P. (2023). Towards understanding centriole elimination. *Open Biol.* 13, 230222. 10.1098/rsob.230222 PMC10645514 37963546

[BIO062609C33] Kathir, P., LaVoie, M., Brazelton, W. J., Haas, N. A., Lefebvre, P. A. and Silflow, C. D. (2003). Molecular map of the *Chlamydomonas reinhardtii* nuclear genome. *Eukaryot. Cell* 2, 362-379. 10.1128/EC.2.2.362-379.2003 PMC154841 12684385

[BIO062609C34] Kindle, K. L. (1990). High-frequency nuclear transformation of *Chlamydomonas reinhardtii*. *Proc. Natl. Acad. Sci. USA* 87, 1228-1232. 10.1073/pnas.87.3.1228 PMC53444 2105499

[BIO062609C35] Kleinhofs, A., Owais, W. M. and Nilan, R. A. (1978). Azide. *Mutat. Res.* 55, 165-195. 10.1016/0165-1110(78)90003-9 107442

[BIO062609C36] Klotz, C., Ruiz, F., Garreau de Loubresse, N., Wright, M., Dupuis-Williams, P. and Beisson, J. (2003). Gamma-tubulin and MTOCs in *Paramecium*. *Protist* 154, 193-209. 10.1078/143446103322166509 13677448

[BIO062609C37] Laporte, M. H., Gambarotto, D., Bertiaux, É., Bournonville, L., Louvel, V., Nunes, J. M., Borgers, S., Hamel, V. and Guichard, P. (2024). Time-series reconstruction of the molecular architecture of human centriole assembly. *Cell* 187, 2158-2174.e19. 10.1016/j.cell.2024.03.025 PMC11060037 38604175

[BIO062609C38] LeGuennec, M., Klena, N., Gambarotto, D., Laporte, M. H., Tassin, A. M., van den Hoek, H., Erdmann, P. S., Schaffer, M., Kovacik, L., Borgers, S. et al. (2020). A helical inner scaffold provides a structural basis for centriole cohesion. *Sci. Adv.* 6, eaaz4137. 10.1126/sciadv.aaz4137 PMC7021493 32110738

[BIO062609C39] LeGuennec, M., Klena, N., Aeschlimann, G., Hamel, V. and Guichard, P. (2021). Overview of the centriole architecture. *Curr. Opin. Struct. Biol.* 66, 58-65. 10.1016/j.sbi.2020.09.015 33176264

[BIO062609C40] Lewin, R. A. (1952). Ultraviolet induced mutations in *Chlamydomonas moewusii* Gerloff. *J. Gen. Microbiol.* 6, 233-248. 10.1099/00221287-6-3-4-233 14927870

[BIO062609C41] Li, J. B., Gerdes, J. M., Haycraft, C. J., Fan, Y., Teslovich, T. M., May-Simera, H., Li, H., Blacque, O. E., Li, L., Leitch, C. C. et al. (2004). Comparative genomics identifies a flagellar and basal body proteome that includes the BBS5 human disease gene. *Cell* 117, 541-552. 10.1016/S0092-8674(04)00450-7 15137946

[BIO062609C42] Li, S., Fernandez, J. J., Marshall, W. F. and Agard, D. A. (2019). Electron cryo-tomography provides insight into procentriole architecture and assembly mechanism. *eLife* 8, e43434. 10.7554/eLife.43434 PMC6384029 30741631

[BIO062609C43] Linck, R. W. and Stephens, R. E. (2007). Functional protofilament numbering of ciliary, flagellar, and centriolar microtubules. *Cell Motil. Cytoskeleton* 64, 489-495. 10.1002/cm.20202 17366641

[BIO062609C44] Liu, P., Würtz, M., Zupa, E., Pfeffer, S. and Schiebel, E. (2021). Microtubule nucleation: The waltz between γ-tubulin ring complex and associated proteins. *Curr. Opin. Cell Biol.* 68, 124-131. 10.1016/j.ceb.2020.10.004 33190097

[BIO062609C45] Matsuura, K., Lefebvre, P. A., Kamiya, R. and Hirono, M. (2004). Bld10p, a novel protein essential for basal body assembly in *Chlamydomonas*: localization to the cartwheel, the first ninefold symmetrical structure appearing during assembly. *J. Cell Biol.* 165, 663-671. 10.1083/jcb.200402022 PMC2172387 15173189

[BIO062609C46] Mukhopadhyay, S., Gao, Q., Manna, T. K. and Schiebel, E. (2026). The γ-TuRC: mastermind of multipurpose actions at the centrosome. *Cell. Mol. Life Sci.* 83, 120. 10.1007/s00018-026-06080-w PMC12932783 41711931

[BIO062609C47] Nakazawa, Y., Hiraki, M., Kamiya, R. and Hirono, M. (2007). SAS-6 is a cartwheel protein that establishes the 9-fold symmetry of the centriole. *Curr. Biol.* 17, 2169-2174. 10.1016/j.cub.2007.11.046 18082404

[BIO062609C48] Nishimura, Y., Higashiyama, T., Suzuki, L., Misumi, O. and Kuroiwa, T. (1998). The biparental transmission of the mitochondrial genome in *Chlamydomonas reinhardtii* visualized in living cells. *Eur. J. Cell Biol.* 77, 124-133. 10.1016/S0171-9335(98)80080-0 9840462

[BIO062609C49] Noga, A., Horii, M., Goto, Y., Toyooka, K., Ishikawa, T. and Hirono, M. (2022). Bld10p/Cep135 determines the number of triplets in the centriole independently of the cartwheel. *EMBO J.* 41, e104582. 10.15252/embj.2020104582 PMC9574746 36093892

[BIO062609C50] Oakley, B. R., Paolillo, V. and Zheng, Y. (2015). γ-Tubulin complexes in microtubule nucleation and beyond. *Mol. Biol. Cell* 26, 2957-2962. 10.1091/mbc.E14-11-1514 PMC4551311 26316498

[BIO062609C51] Osinka, A., Poprzeczko, M., Zielinska, M. M., Fabczak, H., Joachimiak, E. and Wloga, D. (2019). Ciliary proteins: filling the gaps. Recent advances in deciphering the protein composition of motile ciliary complexes. *Cells* 8, 730. 10.3390/cells8070730 PMC6678824 31319499

[BIO062609C52] Pastuglia, M., Azimzadeh, J., Goussot, M., Camilleri, C., Belcram, K., Evrard, J. L., Schmit, A. C., Guerche, P. and Bouchez, D. (2006). Gamma-tubulin is essential for microtubule organization and development in *Arabidopsis*. *Plant Cell* 18, 1412-1425. 10.1105/tpc.105.039644 PMC1475493 16698945

[BIO062609C53] Paz, J. and Lüders, J. (2018). Microtubule-organizing centers: towards a minimal parts list. *Trends Cell Biol.* 28, 176-187. 10.1016/j.tcb.2017.10.005 29173799

[BIO062609C54] Pazour, G. J., Agrin, N., Leszyk, J. and Witman, G. B. (2005). Proteomic analysis of a eukaryotic cilium. *J. Cell Biol.* 170, 103-113. 10.1083/jcb.200504008 PMC2171396 15998802

[BIO062609C55] Ruiz, F., Beisson, J., Rossier, J. and Dupuis-Williams, P. (1999). Basal body duplication in *Paramecium* requires gamma-tubulin. *Curr. Biol.* 9, 43-46. 10.1016/S0960-9822(99)80045-1 9889124

[BIO062609C56] Schweizer, N. and Lüders, J. (2021). From tip to toe – dressing centrioles in γTuRC. *J. Cell Sci.* 134, jcs258397. 10.1242/jcs.258397 34283237

[BIO062609C57] Schweizer, N., Haren, L., Dutto, I., Viais, R., Lacasa, C., Merdes, A. and Lüders, J. (2021). Sub-centrosomal mapping identifies augmin-γTuRC as part of a centriole-stabilizing scaffold. *Nat. Commun.* 12, 6042. 10.1038/s41467-021-26252-5 PMC8519919 34654813

[BIO062609C58] Shang, Y., Li, B. and Gorovsky, M. A. (2002). *Tetrahymena thermophila* contains a conventional gamma-tubulin that is differentially required for the maintenance of different microtubule-organizing centers. *J. Cell Biol.* 158, 1195-1206. 10.1083/jcb.200205101 PMC2173235 12356864

[BIO062609C60] Silflow, C. D., Liu, B., LaVoie, M., Richardson, E. A. and Palevitz, B. A. (1999). Gamma-tubulin in *Chlamydomonas*: characterization of the gene and localization of the gene product in cells. *Cell Motil. Cytoskeleton* 42, 285-297. 10.1002/(SICI)1097-0169(1999)42:4<285::AID-CM3>3.0.CO;2-Z 10223635

[BIO062609C61] Silflow, C. D., LaVoie, M., Tam, L. W., Tousey, S., Sanders, M., Wu, W., Borodovsky, M. and Lefebvre, P. A. (2001). The Vfl1 protein in *Chlamydomonas* localizes in a rotationally asymmetric pattern at the distal ends of the basal bodies. *J. Cell Biol.* 153, 63-74. 10.1083/jcb.153.1.63 PMC2185524 11285274

[BIO062609C62] Sizova, I., Fuhrmann, M. and Hegemann, P. (2001). A *Streptomyces rimosus* aphVIII gene coding for a new type phosphotransferase provides stable antibiotic resistance to *Chlamydomonas reinhardtii*. *Gene* 277, 221-229. 10.1016/S0378-1119(01)00616-3 11602359

[BIO062609C63] Stathatos, G. G., Dunleavy, J. E. M., Zenker, J. and O'Bryan, M. K. (2021). Delta and epsilon tubulin in mammalian development. *Trends Cell Biol.* 31, 774-787. 10.1016/j.tcb.2021.03.010 33867233

[BIO062609C64] Sulimenko, V., Dráberová, E. and Dráber, P. (2022). γ-Tubulin in microtubule nucleation and beyond. *Front. Cell Dev. Biol.* 10, 880761. 10.3389/fcell.2022.880761 PMC9503634 36158181

[BIO062609C65] Sunkel, C. E., Gomes, R., Sampaio, P., Perdigão, J. and González, C. (1995). Gamma-tubulin is required for the structure and function of the microtubule organizing centre in *Drosophila* neuroblasts. *EMBO J.* 14, 28-36. 10.1002/j.1460-2075.1995.tb06972.x PMC398049 7828594

[BIO062609C66] Thawani, A. and Petry, S. (2021). Molecular insight into how γ-TuRC makes microtubules. *J. Cell Sci.* 134, jcs245464. 10.1242/jcs.245464 PMC8325954 34297125

[BIO062609C67] Tovey, C. A. and Conduit, P. T. (2018). Microtubule nucleation by γ-tubulin complexes and beyond. *Essays Biochem.* 62, 765-780. 10.1042/EBC20180028 PMC6281477 30315097

[BIO062609C68] Vásquez-Limeta, A., Lukasik, K., Kong, D., Sullenberger, C., Luvsanjav, D., Sahabandu, N., Chari, R. and Loncarek, J. (2022). CPAP insufficiency leads to incomplete centrioles that duplicate but fragment. *J. Cell Biol.* 221, e202108018. 10.1083/jcb.202108018 PMC9007748 35404385

[BIO062609C69] Wang, J. T. and Stearns, T. (2017). The ABCs of centriole architecture: the form and function of triplet microtubules. *Cold Spring Harb. Symp. Quant. Biol.* 82, 145-155. 10.1101/sqb.2017.82.034496 PMC11156431 29540555

[BIO062609C70] Wieczorek, M., Urnavicius, L., Ti, S. C., Molloy, K. R., Chait, B. T. and Kapoor, T. M. (2020). Asymmetric molecular architecture of the human γ-tubulin ring complex. *Cell* 180, 165-175.e16. 10.1016/j.cell.2019.12.007 PMC7027161 31862189

[BIO062609C71] Wiese, C. and Zheng, Y. (2000). A new function for the gamma-tubulin ring complex as a microtubule minus-end cap. *Nat. Cell Biol.* 2, 358-364. 10.1038/35014051 10854327

[BIO062609C72] Wright, R. L., Chojnacki, B. and Jarvik, J. W. (1983). Abnormal basal-body number, location, and orientation in a striated fiber-defective mutant of C*hlamydomonas reinhardtii*. *J. Cell Biol.* 96, 1697-1707. 10.1083/jcb.96.6.1697 PMC2112458 6853600

[BIO062609C73] Wu, J. and Akhmanova, A. (2017). Microtubule-organizing centers. *Annu. Rev. Cell Dev. Biol.* 33, 51-75. 10.1146/annurev-cellbio-100616-060615 28645217

[BIO062609C74] Yamano, T., Iguchi, H. and Fukuzawa, H. (2013). Rapid transformation of *Chlamydomonas reinhardtii* without cell-wall removal. *J. Biosci. Bioeng.* 115, 691-694. 10.1016/j.jbiosc.2012.12.020 23333644

[BIO062609C75] Yuba-Kubo, A., Kubo, A., Hata, M. and Tsukita, S. (2005). Gene knockout analysis of two gamma-tubulin isoforms in mice. *Dev. Biol.* 282, 361-373. 10.1016/j.ydbio.2005.03.031 15893303

[BIO062609C76] Zheng, X., Ramani, A., Soni, K., Gottardo, M., Zheng, S., Ming Gooi, L., Li, W., Feng, S., Mariappan, A., Wason, A. et al. (2016). Molecular basis for CPAP-tubulin interaction in controlling centriolar and ciliary length. *Nat. Commun.* 7, 11874. 10.1038/ncomms11874 PMC4912634 27306797

[BIO062609C77] Zupa, E., Liu, P., Würtz, M., Schiebel, E. and Pfeffer, S. (2021). The structure of the γ-TuRC: a 25-years-old molecular puzzle. *Curr. Opin. Struct. Biol.* 66, 15-21. 10.1016/j.sbi.2020.08.008 33002806

