# Supplementary material for: Chlamydomonas γ-tubulin mutations reveal a critical role for γ-TuRC in maintaining the stability of centriolar microtubules
Source: Biol Open. 2026 Oct 30;15(11):bio062609. doi: 10.1242/bio.062609 (PMC13750399; doi:10.1242/bio.062609)
Supplement: Supplementary file 1 [file biolopen-15-062609-s1.pdf]

Table S1. Raw data for Table 1: nuclear numbers in wild-type and *bld13* mutants.

Strain		Number of nuclei/cell			Total (n)
		0	1	2 or 3	
wild type	Exp. 1	2 (0.4)	550 (99.6)	0 (0.0)	552
	Exp. 2	4 (0.9)	437 (98.9)	1 (0.2)	442
	Exp. 3	1 (0.3)	330 (99.7)	0 (0.0)	331
	Exp. 4	4 (0.7)	536 (99.1)	1 (0.2)	541
	Exp. 5	3 (0.7)	428 (96.9)	0 (0.0)	431
	Total	14 (0.6)	2281 (99.3)	2 (0.1)	2297
	Mean ± SD (%)	0.6 ± 0.3	99.3 ± 0.4	0.1 ± 0.1	
bld12-1	Exp. 1	13 (3.0)	402 (91.4)	25 (5.7)	440
	Exp. 2	21 (3.4)	560 (89.5)	45 (7.2)	626
	Exp. 3	17 (3.6)	427 (90.7)	27 (5.7)	471
	Exp. 4	27 (4.5)	531 (89.0)	39 (6.5)	597
	Exp. 5	17 (3.9)	398 (91.3)	21 (4.8)	436
	Total	95 (3.7)	2318 (90.2)	157 (6.1)	2570
	Mean ± SD (%)	3.7 ± 0.6	90.3 ± 1.0	6.0 ± 0.9	
bld13-1	Exp. 1	4 (1.0)	377 (95.2)	15 (3.8)	396
	Exp. 2	10 (2.3)	402 (93.5)	18 (4.2)	430
	Exp. 3	5 (1.2)	396 (95.7)	13 (3.1)	414
	Exp. 4	5 (1.2)	411 (94.7)	18 (4.1)	434
	Exp. 5	4 (0.7)	560 (96.9)	14 (2.4)	578
	Total	28 (1.2)	2146 (95.3)	78 (3.4)	2252
	Mean ± SD (%)	1.2 ± 0.6	95.2 ± 1.2	3.5 ± 0.8	
bld13-2	Exp. 1	3 (0.5)	598 (98.8)	4 (0.7)	605
	Exp. 2	6 (0.9)	672 (98.0)	8 (1.2)	686
	Exp. 3	7 (1.2)	565 (97.8)	6 (1.0)	578
	Exp. 4	2 (0.3)	756 (98.6)	9 (1.1)	767
	Exp. 5	3 (0.5)	656 (98.8)	5 (0.8)	664
	Total	21 (0.6)	3247 (98.4)	32 (1.0)	3300
	Mean ± SD (%)	0.7 ± 0.4	98.3 ± 0.5	1.0 ± 0.2	

Biology Open • Supplementary information

Notes: Values indicate the number of cells in each category (% in parentheses). Data represent the results of five independent experiments. Mean ± SD (%) reflect experiment-to-experiment variability and were calculated across independent experiments (Exp.1–Exp.5).

Biology Open • Supplementary information

Table S2. Raw data for Fig. 1 B: number of cells with 0, 1, 2, or ≥3 cilia in wild-type and *bld13* mutants.

Strain		Number of cilia/cell				
		0	1	2	≥3	Total (n)
wild type	Exp. 1	13 (2.9)	2 (0.4)	431 (96.6)	0 (0.0)	446
	Exp. 2	3 (0.8)	4 (1.0)	377 (97.7)	2 (0.5)	386
	Exp. 3	5 (1.2)	4 (1.0)	407 (97.4)	2 (0.5)	418
	Exp. 4	2 (0.5)	5 (1.2)	400 (97.6)	3 (0.7)	410
	Total	23 (1.4)	15 (0.9)	1615 (96.3)	7 (0.4)	1660
	Mean ± SD (%)	1.3 ± 1.1	0.9 ± 0.3	97.3 ± 0.5	0.4 ± 0.3	
bld13-1	Exp. 1	17(6.0)	15(5.3)	251 (88.4)	1 (0.4)	284
	Exp. 2	21(8.4)	23(9.2)	204(81.3)	3 (1.2)	251
	Exp. 3	20 (6.0)	30 (9.0)	280 (84.3)	2 (0.6)	332
	Exp. 4	22 (5.6)	19 (4.9)	235 (88.2)	5 (1.3)	391
	Exp. 5	16 (4.3)	24 (6.5)	328 (88.9)	1 (0.3)	369
	Total	96 (5.9)	111 (6.8)	1408 (86.5)	12 (0.7)	1627
	Mean ± SD (%)	6.1 ± 1.5	7.0 ± 2.0	86.2 ± 3.3	0.7 ± 0.5	
bld13-2	Exp. 1	9 (1.9)	11 (2.3)	454 (95.2)	3 (0.6)	477
	Exp. 2	11 (2.7)	13 (3.2)	374 (93.3)	3 (0.7)	401
	Exp. 3	18 (4.2)	15 (3.5)	394 (92.3)	0 (0.0)	427
	Exp. 4	11 (2.6)	16 (3.8)	396 (93.4)	1 (0.2)	424
	Total	49 (2.83)	55 (3.18)	1618 (93.58)	7 (0.40)	1729
	Mean ± SD (%)	2.9 ± 1.0	3.2 ± 0.6	93.5 ± 1.2	0.4 ± 0.3	

Notes: Values indicate the number of cells in each category (% in parentheses). Data represent the results of four to five independent experiments. Mean ± SD (%) reflect experiment-to-experiment variability and were calculated across independent experiments (Exp.1–Exp.n).

Table S3. Raw data for Fig. 2 A: number of cells with 0, 1, 2, or ≥3 cilia in strains expressing HA-tagged γ-tubulin.

Strain		Number of cilia/cell				
		0	1	2	≥3	Total (n)
WT:: γ-tub(WT)-HA	Exp. 1	8 (2.3)	3 (0.9)	339 (96.9)	0 (0.0)	350
	Exp. 2	9 (2.3)	4 (1.0)	381 (96.7)	0 (0.0)	394
	Exp. 3	7 (1.6)	6 (1.4)	413 (96.7)	1 (0.2)	427
	Exp. 4	5 (1.2)	4 (1.0)	402 (97.6)	1 (0.2)	412
	Total	29 (1.8)	17 (1.1)	1535 (97.0)	2 (0.1)	1583
	Mean ± SD (%)	1.9 ± 0.5	1.1 ± 0.2	97.0 ± 0.4	0.1 ± 0.1	
WT:: γ-tub(T292I)-HA	Exp. 1	29 (7.9)	16 (4.3)	323 (87.5)	1 (0.3)	369
	Exp. 2	13(3.1)	14 (3.3)	391 (93.3)	1 (0.2)	419
	Exp. 3	5 (1.2)	9 (2.2)	394 (96.6)	0 (0.0)	408
	Exp. 4	12 (2.8)	15 (3.4)	406 (93.3)	2 (0.5)	435
	Total	59 (3.6)	54 (3.3)	1514 (92.8)	4 (0.3)	1631
	Mean ± SD (%)	3.7 ± 2.9	3.3 ± 0.9	92.7 ± 3.8	0.2 ± 0.2	
WT:: γ-tub(E89D)-HA	Exp. 1	12 (1.9)	12 (2.3)	365 (95.2)	3 (0.6)	392
	Exp. 2	6 (2.7)	10 (3.2)	388 (93.3)	2 (0.7)	406
	Exp. 3	13 (4.2)	8 (3.5)	391 (92.3)	2 (0.0)	414
	Exp. 4	4 (2.6)	9 (3.8)	302 (93.4)	2 (0.2)	317
	Total	35 (2.3)	39 (2.6)	1446 (94.6)	9 (0.6)	1529
	Mean ± SD (%)	2.2 ± 1.0	2.6 ± 0.5	94.6 ± 1.1	0.6 ± 0.1	
bld13-1:: γ-tub(WT)-HA	Exp. 1	10 (2.8)	14 (3.9)	332 (93.0)	1 (0.3)	357
	Exp. 2	8(1.9)	22 (5.1)	397 (91.9)	5 (1.2)	432
	Exp. 3	8 (2.1)	24 (6.3)	348 (91.1)	2 (0.5)	382
	Exp. 4	10 (1.7)	24 (4.0)	564 (93.5)	5 (0.8)	603
	Total	36 (2.0)	84 (4.7)	1641 (92.5)	13 (0.7)	1774
	Mean ± SD (%)	2.1 ± 0.5	4.8 ± 1.1	92.4 ± 0.5	0.7 ± 0.4	
bld13-2:: γ-tub(WT)-HA	Exp. 1	13 (2.9)	23 (5.1)	411 (91.3)	3 (0.7)	450
	Exp. 2	13 (4.3)	17 (5.7)	265 (89.2)	2 (0.7)	297
	Exp. 3	16 (3.7)	17 (3.9)	399 (91.9)	2 (0.5)	434
	Exp. 4	20 (4.7)	14 (3.3)	384 (91.4)	2 (0.5)	420
	Total	62 (3.9)	71 (4.4)	1459 (91.1)	9 (0.6)	1601
	Mean ± SD (%)	3.9 ± 0.8	4.5 ± 1.1	91.0 ± 1.2	0.6 ± 0.1	

Biology Open • Supplementary information

Notes: Values indicate the number of cells in each category (% in parentheses). Data represent the results of four independent experiments. Mean ± SD (%) reflect experiment-to-experiment variability and were calculated across independent experiments (Exp.1–Exp.4).

Table S4. Raw data for Fig. 2 C: number of cells with 0, 1, 2, or ≥3 cilia in diploid strains.

Strain		Number of cilia/cell				Total (n)
		0	1	2	≥3	
WT/WT	Exp. 1	25 (6.9)	11 (3.0)	325 (89.8)	1 (0.3)	362
	Exp. 2	33 (9.8)	7 (2.1)	294 (87.2)	3 (0.9)	337
	Exp. 3	31 (8.9)	7 (2.0)	310 (88.8)	1 (0.3)	349
	Exp. 4	34 (9.6)	6 (1.7)	313 (88.2)	2 (0.6)	355
	Exp. 5	34 (6.3)	6 (1.1)	491 (92.3)	1 (0.2)	532
	Total	157 (8.1)	37 (1.9)	1733 (89.6)	8 (0.4)	1935
	Mean ± SD (%)	8.3 ± 1.6	2.0 ± 0.7	89.3 ± 1.9	0.4 ± 0.3	
WT/*bld13-1*	Exp. 1	55 (15.0)	24 (6.5)	284 (77.4)	4 (1.1)	367
	Exp. 2	67 (17.3)	25 (6.5)	294 (76.0)	1 (0.3)	387
	Exp. 3	56 (14.7)	25 (6.6)	298 (78.4)	1 (0.3)	380
	Exp. 4	64 (16.1)	17 (4.3)	314 (79.1)	2 (0.5)	397
	Exp. 5	75 (19.2)	24 (6.2)	290 (74.3)	1 (0.3)	390
	Total	317 (16.5)	115 (6.0)	1480 (77.0)	9 (0.5)	1921
	Mean ± SD (%)	16.5 ± 1.8	6.0 ± 1.0	77.0 ± 1.9	0.5 ± 0.4	
WT/*bld13-2*	Exp. 1	41 (10.5)	18 (4.6)	328 (84.3)	2 (0.5)	389
	Exp. 2	59 (15.9)	13 (3.5)	297 (80.3)	1 (0.3)	370
	Exp. 3	41 (11.5)	20 (5.6)	290 (81.7)	4 (1.1)	355
	Exp. 4	57 (13.1)	17 (3.9)	360 (82.6)	2 (0.5)	436
	Exp. 5	56 (14.9)	25 (6.7)	293 (78.1)	1 (0.3)	375
	Total	254 (13.2)	93 (4.8)	1568 (81.5)	10 (0.5)	1925
	Mean ± SD (%)	13.2 ± 2.3	4.8 ± 0.7	81.4 ± 2.3	0.5 ± 0.4	
bld13-1/bld13-2	Exp. 1	49 (13.1)	25 (6.7)	292 (78.3)	7 (1.9)	373
	Exp. 2	110 (25.0)	27 (6.1)	298 (67.6)	6 (1.4)	441
	Exp. 3	81 (19.5)	20 (4.8)	308 (74.2)	6 (1.4)	415
	Exp. 4	74 (16.2)	24 (5.3)	353 (77.2)	6 (1.3)	457
	Exp. 5	85 (20.7)	20 (4.9)	303 (73.7)	3 (0.7)	411
	Total	399 (19.0)	116 (5.5)	1554 (74.1)	28 (1.3)	2097
	Mean ± SD (%)	18.9 ± 4.5	5.5 ± 0.8	74.2 ± 4.2	1.3 ± 0.4	

Notes: Values indicate the number of cells in each category (% in parentheses). Data represent the results of five independent experiments. Mean ± SD (%) reflect experiment-to-experiment variability and were calculated across independent experiments (Exp.1–Exp.5).

Table S5. Raw data for Fig. 4: cell counts for each category of microtubule organization phenotype.

		Number of microtubule/cell				
Antibody	Strain	3≤	4	≥5	CL	Total (n)
Anti-acetylated α-tubulin	wild type	6 (3.3)	111 (60.7)	66 (36.1)	0 (0.0)	183
	*bld13-1******	0 (0.0)	34 (22.8)	110 (73.8)	5 (3.4)	149
	*bld13-2******	3 (2.4)	50 (40.0)	69 (55.2)	3 (2.4)	125
Anti-α-tubulin	wild type	7 (2.9)	85 (35.4)	148 (61.7)	0 (0.0)	240
	*bld13-1******	6 (3.0)	42 (21.1)	140 (70.4)	11 (5.5)	199
	*bld13-2*ns	3 (3.9)	70 (38.7)	103 (56.9)	1 (0.6)	181

Notes: Cells were classified into four categories based on number of microtubule per cell. Values indicate the number of cells in each category (% in parentheses). *P*-values were calculated using Fisher's exact test for the overall distribution of microtubule organization (****P* < 0.001, ns, not significant vs wild type).

Biology Open • Supplementary information

Table S6. Raw data for Fig. 5 C: distribution of protofilament loss in A- and C-tubules of triplet microtubules.

Strain		Sector number 1	2	3	4	5	6	7	8	9	10	11	12	Total triplet number
bld13-1	A-tubule	6 (3.8)	4 (2.5)	0 (0.0)	0 (0.0)	0 (0.0)	0 (0.0)	0 (0.0)	0 (0.0)	10 (6.3)	20 (12.7)	31 (19.6)	17 (10.8)	158
	C-tubule	0 (0.0)	0 (0.0)	3 (5.3)	6 (10.5)	6 (10.5)	6 (10.5)	—	—	—	—	—	—	57
bld13-2	A-tubule	1 (0.5)	0 (0.0)	0 (0.0)	0 (0.0)	0 (0.0)	0 (0.0)	0 (0.0)	0 (0.0)	3 (1.4)	9 (4.2)	18 (8.5)	10 (4.7)	212
	C-tubule	0 (0.0)	1 (1.1)	18 (19.6)	18 (19.6)	13 (14.1)	12 (13.0)	—	—	—	—	—	—	92

Notes: Values indicate the number of cells in each category (% in parentheses). Percentages represent the frequency of protofilament loss in each sector relative to total triplets; multiple losses per triplet were counted independently. Statistical significance was assessed as described in the Figure 5 legend.

Table S7. Raw data for Fig. S3: distribution of protofilament loss in A- and C-tubules of triplet microtubules in independent replicates of *bld13* mutants, with additional data for diploid *bld13-1/bld13-2*.

Strain			Sector number												Total triplet number
			1	2	3	4	5	6	7	8	9	10	11	12	
bld13-1	A-tubule	#1	4 (3.3)	0 (0.0)	0 (0.0)	0 (0.0)	0 (0.0)	0 (0.0)	0 (0.0)	0 (0.0)	7 (5.7)	5 (4.1)	9 (7.3)	10 (8.1)	123
		#2	7 (4.4)	0 (0.0)	0 (0.0)	0 (0.0)	0 (0.0)	0 (0.0)	0 (0.0)	1 (0.6)	3 (1.9)	3 (1.9)	5 (3.1)	5 (3.1)	159
		Total	11 (3.9)	0 (0.0)	0 (0.0)	0 (0.0)	0 (0.0)	0 (0.0)	0 (0.0)	1 (0.4)	10 (3.5)	8 (2.8)	14 (5.0)	15 (5.3)	282
	C-tubule	#1	0 (0.0)	1 (2.6)	3 (7.7)	5 (12.8)	2 (5.1)	1 (2.6)	—	—	—	—	—	—	39
		#2	0 (0.0)	1 (1.5)	1 (1.5)	4 (5.9)	2 (2.9)	1 (1.5)	—	—	—	—	—	—	68
		Total	0 (0.0)	2 (1.9)	4 (3.7)	9 (8.4)	4 (3.7)	2 (1.9)	—	—	—	—	—	—	107
bld13-2	A-tubule	#1	5 (3.2)	0 (0.0)	0 (0.0)	0 (0.0)	0 (0.0)	0 (0.0)	0 (0.0)	0 (0.0)	1 (0.6)	5 (3.2)	9 (5.7)	10 (6.4)	157
		#2	2 (1.2)	0 (0.0)	0 (0.0)	0 (0.0)	0 (0.0)	0 (0.0)	0 (0.0)	1 (1.2)	3 (1.8)	5 (3.0)	5 (3.0)	3 (1.8)	167
		Total	7 (2.5)	0 (0.0)	0 (0.0)	0 (0.0)	0 (0.0)	0 (0.0)	0 (0.0)	1 (0.4)	4 (1.4)	10 (3.5)	14 (5.0)	13 (4.6)	324
	C-tubule	#1	0 (0.0)	1 (2.4)	4 (9.5)	6 (14.3)	5 (11.9)	2 (4.8)	—	—	—	—	—	—	42
		#2	0 (0.0)	2 (4.7)	2 (4.7)	2 (4.7)	3 (7.0)	1 (2.3)	—	—	—	—	—	—	43
		Total	0 (0.0)	3 (3.5)	6 (7.1)	8 (9.4)	8 (9.4)	3 (3.5)	—	—	—	—	—	—	85
bld13-1 /bld13-2	A-tubule	#1	7 (6.3)	1 (0.9)	0 (0.0)	0 (0.0)	0 (0.0)	0 (0.0)	0 (0.0)	1 (0.9)	4 (3.6)	4 (3.6)	8 (7.2)	12 (10.8)	111
		#2	5 (4.1)	1 (0.8)	0 (0.0)	0 (0.0)	0 (0.0)	0 (0.0)	0 (0.0)	1 (0.8)	4 (3.3)	8 (6.6)	9 (7.4)	7 (5.7)	122
		Total	12 (5.2)	2 (0.9)	0 (0.0)	0 (0.0)	0 (0.0)	0 (0.0)	0 (0.0)	2 (0.9)	8 (3.4)	12 (5.2)	17 (7.3)	19 (8.2)	233
	C-tubule	#1	0 (0.0)	0 (0.0)	2 (6.5)	5 (16.1)	3 (9.7)	2 (6.5)	—	—	—	—	—	—	31
		#2	0 (0.0)	2 (4.7)	3 (7.0)	2 (4.7)	3 (7.0)	2 (4.7)	—	—	—	—	—	—	43
		Total	0 (0.0)	2 (2.7)	5 (6.8)	7 (9.5)	6 (8.1)	4 (5.4)	—	—	—	—	—	—	

Notes: Values indicate the number of cells in each category (% in parentheses).

Percentages represent the frequency of protofilament loss in each sector relative to total

Biology Open • Supplementary information

triplets; multiple losses per triplet were counted independently. Data were obtained from two independent biological replicates for each genotype. Data from haploid *bld13* mutants represent independent replicates of the analysis shown in Fig. 5C. Statistical significance was assessed as described in the Fig. S3 legend.

Biology Open • Supplementary information

Table S8. Frequencies of triplet images with partial protofilament loss (%) in two biological replicates for wild type, *bld13-1*, and *bld13-2*.

Strain			Regions in longitudinal axis of the centriole (%)			
			Transition zone	Distal	Central	Proximal
wild type	A-tubule	#1	ND	0/63 (0.0)	1/57 (1.8)	0/39 (0.0)
		#2	ND	0/55 (0.0)	0/41 (0.0)	0/32 (0.0)
	C-tubule	#1	ND	ND	0/31 (0.0)	ND
		#2	ND	ND	0/35 (0.0)	ND
bld13-1	A-tubule	#1	0/41 (0.0)	1/32 (6.7)	9/58 (15.5)	7/33 (21.2)
		#2	0/52 (0.0)	0/32 (0.0)	8/90 (8.9)	7/37 (18.9)
	C-tubule	#1	ND	ND	6/39 (15.4)	ND
		#2	ND	ND	5/68 (7.4)	ND
bld13-2	A-tubule	#1	0/34 (0.0)	0/51 (0.0)	7/66 (10.6)	6/40 (15.0)
		#2	0/39 (0.0)	1/61 (1.6)	6/63 (9.5)	5/43 (11.6)
	C-tubule	#1	ND	ND	8/42 (19.0)	ND
		#2	ND	ND	6/43 (14.0)	ND

Notes: Percentages indicate the proportion of observed triplet microtubule images showing loss of one or more protofilaments. Values are given as *n/N*, where *n* is the number of triplets with protofilament loss and *N* is the total number of triplet microtubules analyzed from electron microscopy images. Data were obtained from two independent biological replicates. ND, not determined.

Biology Open • Supplementary information

Table S9. Frequencies of triplet images with partial protofilament loss (%) in two biological replicates for diploid strain WT/WT and *bld13-1*/*bld13-2*.

Strain			Regions in longitudinal axis of the centriole (%)			
			Transition zone	Distal	Central	Proximal
WT/WT	A-tubule	#1	ND	0/63 (0.0)	0/57 (0.0)	0/23 (0.0)
		#2	ND	0/55 (0.0)	0/45 (0.0)	0/40 (0.0)
	C-tubule	#1	ND	ND	1/51 (2.0)	ND
		#2	ND	ND	0/38 (0.0)	ND
bld13-1/bld13-2	A-tubule	#1	0/32 (0.0)	2/26 (7.7)	10/52 (19.2)	8/33 (24.2)
		#2	0/30 (0.0)	2/34 (5.9)	9/52 (17.3)	9/36 (25.0)
	C-tubule	#1	ND	ND	5/31 (16.1)	ND
		#2	ND	ND	6/43 (14.0)	ND

Notes: Percentages indicate the proportion of observed triplet microtubule images showing loss of one or more protofilaments. Values are given as *n/N*, where *n* is the number of triplets with protofilament loss and *N* is the total number of triplet microtubules analyzed from electron microscopy images. Data were obtained from two independent biological replicates. ND, not determined.

Biology Open • Supplementary information

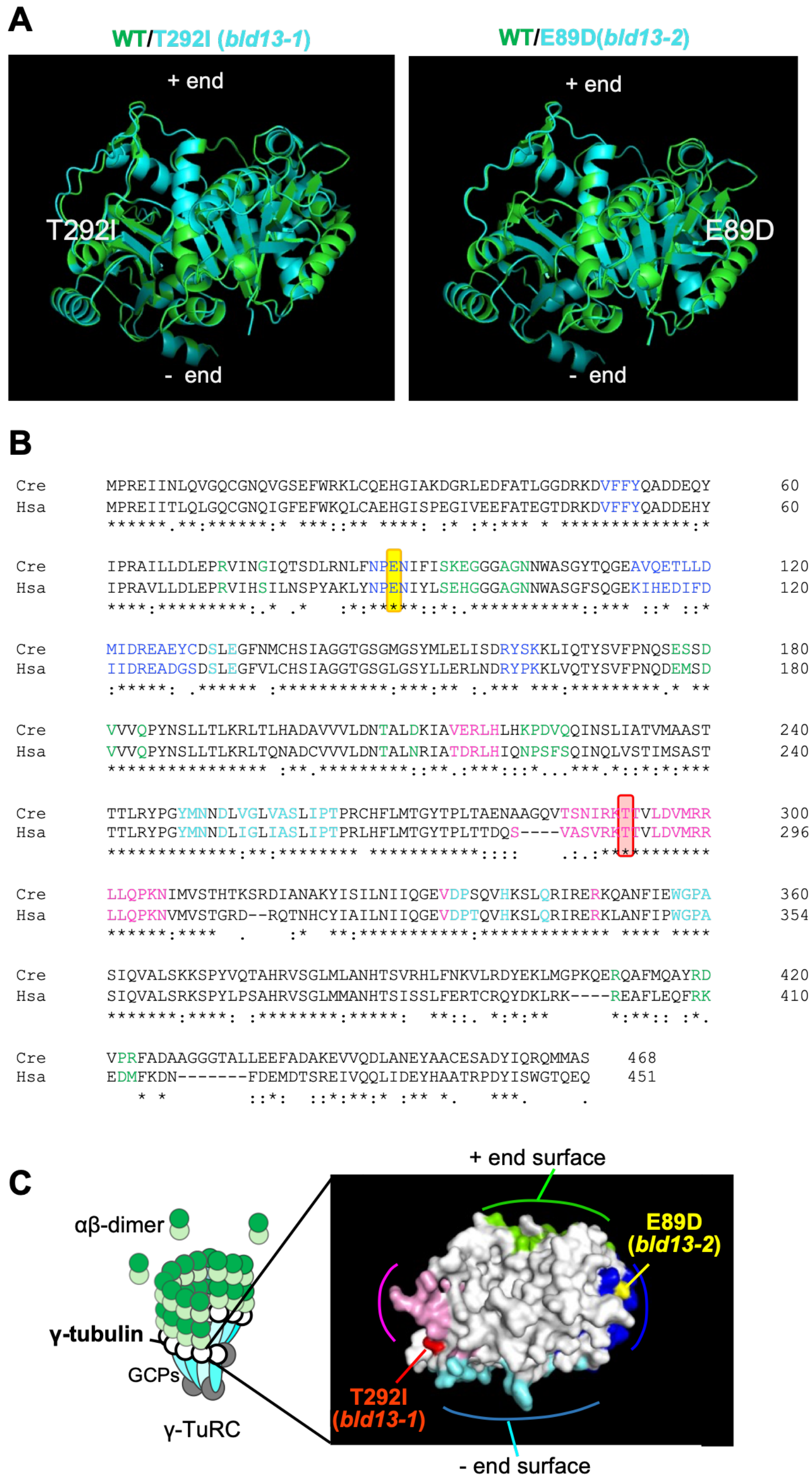

Fig. S1. Effects of the *bld13* mutations on the γ-tubulin structure predicted by AlphaFold2. (A) AlphaFold2 models of the *bld13-1* and *bld13-2* γ-tubulin molecules (blue) are merged with that of the wild-type (green). The mutation sites (T292I and E89D) are shown in white. (B) An amino acid sequence alignment of human γ-tubulin

Biology Open • Supplementary information

(Acc. No. NP_001061.2) and *Chlamydomonas* γ-tubulin (Acc. No. AAA82610.1). Amino acids homologous or conserved between the two proteins were identified by Clustal Omega (Sievers and Higgins, 2014) and indicated by an asterisk or dot, respectively. Indicated by color are the amino acids located on the + end surface (green) and the - end surface (light blue), and those close to the adjacent γ-tubulin molecules (blue and pink). The positions of the mutated amino acids (E89 and T292) are indicated with yellow and red boxes. Note that they are one of the amino acids located close to the adjacent γ-tubulin. (C) Positions of the amino acid changes on the γ-tubulin 3-D structure. A model of the human γ-tubulin 3-D structure, based on the PDB file 1Z5W (Aldaz et al., 2005), is generated using PyMOL (right). This model shows the surface of the γ-tubulin molecule in the same orientation as in the γ-TuRC (left). The colors correspond to those in (B).

Biology Open • Supplementary information

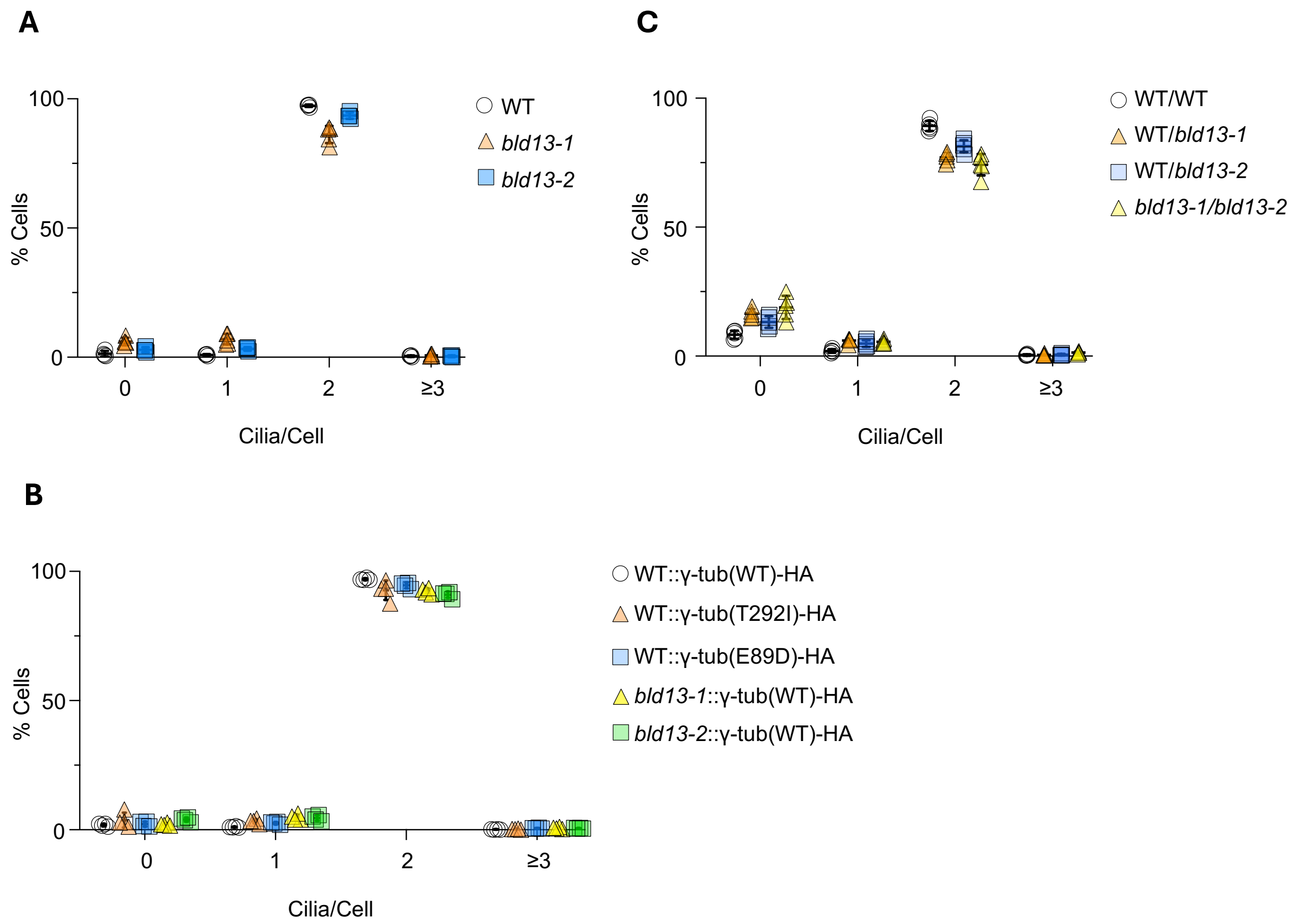

Fig. S2. Dot plots showing individual data points from independent experiments corresponding to Fig. 1B, 2A, and 2C. (A–C) Data corresponding to Fig. 1B, 2A, and 2C, respectively. Each point represents the percentage of cells with 0, 1, 2, or ≥3 cilia in a given experiment. Horizontal bars and error bars represent the mean and standard deviation (SD), respectively. Raw counts and mean ± SD values for each cilia number category are provided in Tables S2–S4.

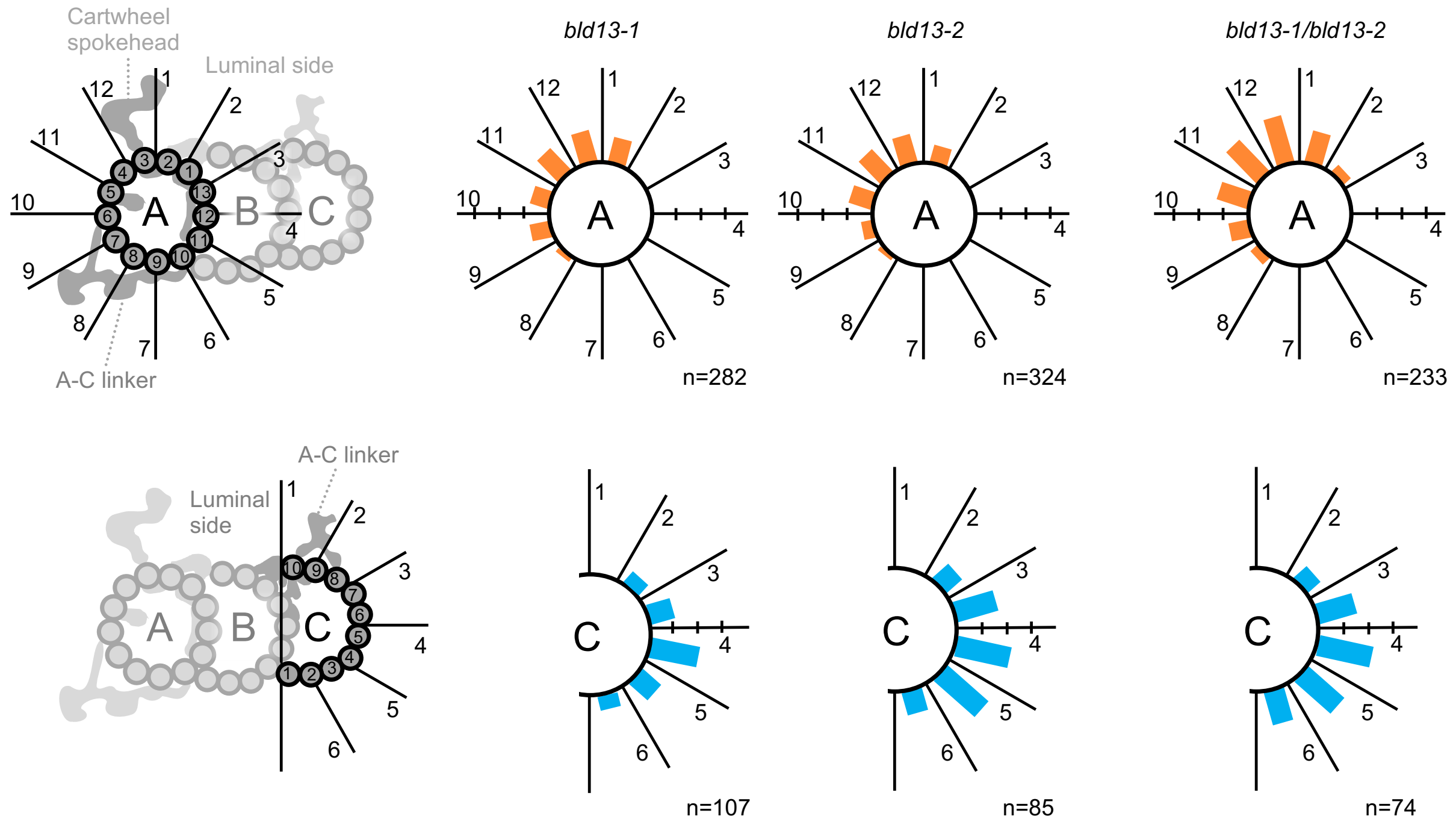

Fig. S3. Independent biological replicates of protofilament loss distributions in A- and C-tubules of *bld13-1* and *bld13-2* triplet microtubules, and corresponding data for the diploid strain *bld13-1/bld13-2*. All data shown in this figure were obtained independently of those shown in Fig. 5C. Sector definitions and plotting conventions are as in Fig. 5C (see Materials and Methods). For each strain, cells were cultured and fixed independently on two separate occasions, and counts from both preparations were pooled for each polar histogram. The length of each bar represents the percentage of protofilament loss at each sector relative to the total number of observed triplet microtubules. The x-axis of each graph is shown in 5% increments. Statistical analysis was performed using the Hodges–Ajne test, showing that all distributions of the protofilament loss are significantly non-uniform: *bld13-1* A-tubule ($P < 0.0001$; median: sector 11), *bld13-2* A-tubule ($P < 0.0001$; median: sector 11), *bld13-1/bld13-2* A-tubule ($P < 0.0001$; median: sector 11), *bld13-1* C-tubule ($P < 0.0001$; median: sector 4), *bld13-2*

C-tubule ($P < 0.0001$; median: sector 4), *bld13-1*/*bld13-2* C-tubule ($P < 0.0001$; median: sector 4). Raw counts underlying these distributions are provided in Table S7.

References

Aldaz H., Rice L.M., Stearns T., and Agard D.A. (2005). Insights into microtubule nucleation from the crystal structure of human gamma-tubulin. *Nature* **435**, 523-527. doi: 10.1038/nature03586.

Sievers F., and Higgins D.G. (2014). Clustal omega. *Curr. Protoc. Bioinformatics* **48**, 3.13.1-3.13.16. doi: 10.1002/0471250953.bi0313s48.
